# Cytokine/Chemokine Release Patterns and Transcriptomic Profiles of LPS/IFNγ-Activated Human Macrophages Differentiated with Heat-Killed *Mycobacterium obuense*, M-CSF, or GM-CSF

**DOI:** 10.3390/ijms22137214

**Published:** 2021-07-05

**Authors:** Samer Bazzi, Emale El-Darzi, Tina McDowell, Helmout Modjtahedi, Satvinder Mudan, Marcel Achkar, Charles Akle, Humam Kadara, Georges M. Bahr

**Affiliations:** 1 Division of Biology and Environmental Science, Faculty of Arts and Sciences, University of Balamand, 33 Amioun, Al Kurah 100, Lebanon; 2 Department of Biomedical Sciences, Faculty of Medicine and Medical Sciences, University of Balamand, 33 Amioun, Al Kurah 100, Lebanon; emaledarzi@gmail.com (E.E.-D.); georges.bahr@balamand.edu.lb (G.M.B.); 3 Department of Translational Molecular Pathology, The University of Texas MD Anderson Cancer Center, Houston, TX 77030, USA; CLMcDowell@mdanderson.org (T.M.); HKadara@mdanderson.org (H.K.); 4 School of Life Sciences, Pharmacy and Chemistry, Faculty of Science, Engineering and Computing, Kingston University, Kingston upon Thames, Surrey KT1 2EE, UK; H.Modjtahedi@kingston.ac.uk; 5 The London Clinic, 20 Devonshire Pl, London W1G 6BW, UK; mudans@aol.com (S.M.); cakle@me.com (C.A.); 6 Clinical Laboratory, Nini Hospital, Tripoli 1434, Lebanon; marcel.achkar@hopitalnini.com

**Keywords:** *Mycobacterium obuense*, immunomodulation, monocyte-derived macrophages, macrophage activation, RNA sequencing

## Abstract

Macrophages (Mφs) are instrumental regulators of the immune response whereby they acquire diverse functional phenotypes following their exposure to microenvironmental cues that govern their differentiation from monocytes and their activation. The complexity and diversity of the mycobacterial cell wall have empowered mycobacteria with potent immunomodulatory capacities. A heat-killed (HK) whole-cell preparation of *Mycobacterium obuense* (*M. obuense*) has shown promise as an adjunctive immunotherapeutic agent for the treatment of cancer. Moreover, HK *M. obuense* has been shown to trigger the differentiation of human monocytes into a monocyte-derived macrophage (MDM) type named Mob-MDM. However, the transcriptomic profile and functional properties of Mob-MDMs remain undefined during an activation state. Here, we characterized cytokine/chemokine release patterns and transcriptomic profiles of lipopolysaccharide (LPS)/interferon γ (IFNγ)-activated human MDMs that were differentiated with HK *M. obuense* (Mob-MDM(LPS/IFNγ)), macrophage colony-stimulating factor M-MDM(LPS/IFNγ)), or granulocyte/macrophage colony-stimulating factor (GM-MDM(LPS/IFNγ)). Mob-MDM(LPS/IFNγ) demonstrated a unique cytokine/chemokine release pattern (interleukin (IL)-10^low^, IL-12/23p40^low^, IL-23p19/p40^low^, chemokine (C-x-C) motif ligand (CXCL)9^low^) that was distinct from those of M-MDM(LPS/IFNγ) and GM-MDM(LPS/IFNγ). Furthermore, M-MDM(LPS/IFNγ) maintained IL-10 production at significantly higher levels compared to GM-MDM(LPS/IFNγ) and Mob-MDM(LPS/IFNγ) despite being activated with M1-Mφ-activating stimuli. Comparative RNA sequencing analysis pointed to a distinct transcriptome profile for Mob-MDM(LPS/IFNγ) relative to both M-MDM(LPS/IFNγ) and GM-MDM(LPS/IFNγ) that comprised 417 transcripts. Functional gene-set enrichment analysis revealed significant overrepresentation of signaling pathways and biological processes that were uniquely related to Mob-MDM(LPS/IFNγ). Our findings lay a foundation for the potential integration of HK *M. obuense* in specific cell-based immunotherapeutic modalities such as adoptive transfer of Mφs (Mob-MDM(LPS/IFNγ)) for cancer treatment.

## 1. Introduction

Macrophages (Mφs) are known for their exceptional degree of plasticity whereby they display diversified phenotypes and functions following exposure to various cues from the surrounding milieu [1]. Human Mφs are generally classified into “proinflammatory/classically activated” M1 and “anti-inflammatory/alternatively activated” M2 phenotypes. M1-Mφs produce significant amounts of proinflammatory cytokines (e.g., interleukin (IL)-1β, IL-6, IL-12, IL-23, and tumor necrosis factor alpha (TNF-α)) and retain robust and antitumor capabilities in certain stages of cancer development and progression. In contrast, M2-Mφs release substantial amounts of anti-inflammatory cytokines (e.g., IL-10 and transforming growth factor (TGF-β)) and contribute to tissue regeneration, angiogenesis, and tumor progression [2,3]. To obtain activated human Mφ phenotypes in vitro, isolated monocytes are initially primed with maturation/differentiation factors such as granulocyte/macrophage colony-stimulating factor (GM-CSF) and macrophage colony-stimulating factor (M-CSF) to generate nonactivated M1-like and M2-like monocyte-derived macrophages (MDMs), respectively [4,5,6]. Following the maturation or differentiation stage, M1-like Mφs are stimulated with lipopolysaccharide (LPS) and interferon γ (IFNγ) to induce the activated M1-Mφ phenotype, while M2-like Mφs are stimulated with interleukin (IL)-4 ± IL-13, IL-10, TGF-β, immune complexes, or glucocorticoids to induce various activated M2-Mφ phenotypes [7,8]. 

The complexity and diversity of the mycobacterial cell wall have empowered mycobacteria with a potent immunomodulatory capacity [9]. Such mycobacteria-associated immunomodulatory activities have been mainly attributed to various mycobacterial cell wall components, serving as pathogen-associated molecular patterns (PAMPs) that are recognized by specific pathogen recognition receptors (PRRs) expressed on various innate immune cells, including monocytes, and macrophages [10,11]. Over the past 10 years, there has been an increasing interest in exploring the immunotherapeutic potential [12,13,14] and the in vitro immunomodulatory properties of a heat-killed (HK) whole-cell preparation of *Mycobacterium obuense* (*M. obuense*), also known as IMM-101 [15,16,17,18]. In a phase I clinical trial, HK *M. obuense* was shown to be safe and well tolerated when used alone in patients with stage III/IV metastatic melanoma [12]. A phase II clinical trial reported that a combination treatment of HK *M. obuens*e and conventional gemcitabine chemotherapy was safe and resulted in a significant increase in the overall survival of patients with metastatic pancreatic cancer [13]. HK *M. obuense* is currently being investigated as an immunotherapeutic agent in combination with anti-PD-1 in a phase II trial (NCT03711188) involving patients with advanced melanoma. In addition, an ongoing phase III clinical trial (NCT04442048) is examining whether immunization with HK *M. obuense* can diminish the frequency of severe respiratory and severe acute respiratory syndrome coronavirus 2 (SARS-CoV-2)-related infections among cancer patients. Our group previously demonstrated the ability of HK *M. obuense* to modulate the surface expression of different categories of cells surface receptors on whole-blood human monocytes [16], as well as to trigger the differentiation of human monocytes into a nonactivated Mφ type (Mob-MDM) that is quite distinct from the nonactivated Mφ phenotypes generated by M-CSF (M-MDM) or GM-CSF (GM-MDM) [18]. Nonactivated Mob-MDMs exhibited significantly increased and spontaneous release of the proinflammatory cytokines IL-6 and TNF-α and chemokine (C-x-C) motif ligand (CXCL) 8, relative to nonactivated M-MDMs and GM-MDMs. Moreover, all three nonactivated Mφ types did not release spontaneous detectable levels of IL-10, IL-12p40, IL-12p70, and IL-23p19/p40. In the same study, genome-wide gene expression profiling combined with functional enrichment analysis revealed unique gene sets and networks suggestive of a proinflammatory M1-like Mφ phenotype in Mob-MDMs compared with both M-MDMs and GM-MDMs [18].

Because human nonactivated MDMs display a distinct pattern of phenotypic and functional characteristics compared to activated MDMs [19,20], this study sought to characterize M-MDMs, GM-MDMs, and Mob-MDMs during an activated state. Therefore, the three above-mentioned nonactivated MDM types were activated with LPS and IFNγ to generate the following MDM types: M-MDM(LPS/IFNγ), GM-MDM(LPS/IFNγ), and Mob-MDM(LPS/IFNγ), whereby the Mφ nomenclature proposed by Murray et al. was adopted [1]. The aims of this study were, first, to characterize the cytokine and chemokine secretion patterns of M-MDM(LPS/IFNγ), GM-MDM(LPS/IFNγ), and Mob-MDM(LPS/IFNγ) and, second, to perform genome-wide transcriptome profiling of the three LPS/IFNγ-activated MDM types.

## 2. Results

### 2.1. Patterns of Cytokine and Chemokine Secretion by M-MDM(LPS/IFNγ), GM-MDM(LPS/IFNγ), and Mob-MDM(LPS/IFNγ)

**Figure 1:**
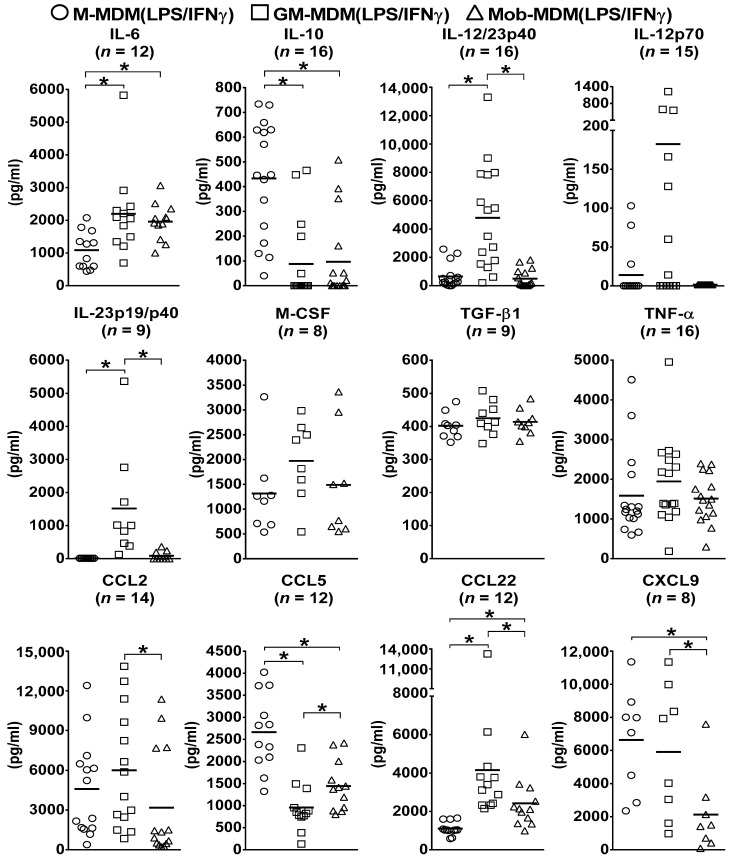
Cytokine and chemokine secretion by lipopolysaccharide (LPS)/interferon γ (IFNγ)-activated monocyte-derived macrophages (MDMs). Macrophage colony-stimulating factor MDMs (M-MDMs), granulocyte/macrophage colony-stimulating factor MDMs (GM-MDM), and heat-killed *Mycobacterium obuense* MDMs (Mob-MDMs) were generated and activated with LPS and IFNγ for 24 h as described below (see the Materials and Methods section (Section 4)). Cytokine and chemokine levels were determined in M-MDM(LPS/IFNγ), GM-MDM(LPS/IFNγ), and Mob-MDM(LPS/IFNγ) culture supernatants by ELISA. Scatter plots demonstrate chemokine or cytokine concentration (pg/mL) in different LPS/IFNγ-activated MDM culture supernatants. Horizontal bars indicate group mean values of cytokine or chemokine concentration of at least 8 independent donors. One-way ANOVA, followed by Tukey’s post-hoc test, was performed to determine statistically significant differences in cytokine/chemokine secreted levels (* *p* < 0.05). Circles represent M-MDM(LPS/IFNγ), squares represent GM-MDM(LPS/IFNγ) and triangles represent Mob-MDM(LPS/IFNγ).

To examine the functional characteristics of different LPS/IFNγ-activated MDMs, culture supernatants of M-MDM(LPS/IFNγ), GM-MDM(LPS/IFNγ), and Mob-MDM(LPS/IFNγ) were screened for various secreted cytokines and chemokines. GM-MDM(LPS/IFNγ) displayed significantly (*p* < 0.05) higher secretion levels of the M1-Mφ signature cytokine, IL-12/23p40, as compared to M-MDM(LPS/IFNγ) and Mob-MDM(LPS/IFNγ) (~7.5-fold and ~10-fold, respectively; Figure 1). Of note, GM-MDM(LPS/IFNγ) were found to release substantial amounts of another M1-Mφ marker, IL-23p19/p40, while M-MDM(LPS/IFNγ) and Mob-MDM(LPS/IFNγ) were characterized by their undetectable and low-level release of IL-23p19/p40, respectively (Figure 1). Additionally, GM-MDM(LPS/IFNγ) showed a trend toward higher IL-12p70 production relative to M-MDM(LPS/IFNγ) and Mob-MDM(LPS/IFNγ); however, this difference did not achieve statistical significance (Figure 1). Supernatants from M-MDM(LPS/IFNγ) cultures demonstrated significantly (*p* < 0.05) increased levels of chemokine (C-C motif) ligand 5 (CCL5) and the M2-Mφ signature cytokine marker, IL-10, as well as decreased levels of M1-Mφ marker, IL-6, when compared with supernatants from GM-MDM(LPS/IFNγ) and Mob-MDM(LPS/IFNγ) cultures (Figure 1). In addition, Mob-MDM(LPS/IFNγ) exhibited significantly (*p* < 0.05) reduced CXCL9 secretion as compared to M-MDM(LPS/IFNγ) and GM-MDM(LPS/IFNγ) (Figure 1). Mob-MDM(LPS/IFNγ) released lower amounts of CCL2 and CCL22 as compared to GM-MDM(LPS/IFNγ). No significant differences were detected between M-MDM(LPS/IFNγ), GM-MDM(LPS/IFNγ), and Mob-MDM(LPS/IFNγ) in terms of TGF-β1, TNF-α, and M-CSF production (Figure 1). All in all, the above-reported data strongly indicate that Mob-MDM(LPS/IFNγ) are characterized by a distinct cytokine and chemokine secretion pattern (IL-10^low^, IL-12/23p40^low^, IL-23p19/p40^low^, and CXCL9^low^) from that of M-MDM(LPS/IFNγ) and GM-MDM(LPS/IFNγ). Moreover, the cytokine release patterns of GM-DM(LPS/IFNγ) and M-MDM(LPS/IFNγ) strongly correlate with those of M1-Mφs and M2-Mφs, respectively. The cytokine and chemokine secretion profiles of nonactivated M-MDMs, GM-MDMs, and Mob-MDMs have been characterized in a previous study [18].

### 2.2. Comparative Whole-Transcriptome Analysis of M-MDM(LPS/IFNγ), GM-MDM(LPS/IFNγ), and Mob-MDM(LPS/IFNγ)

RNA sequencing (RNA-Seq) was carried out to profile the transcriptomes of LPS/IFNγ-activated Mob-MDMs, M-MDMs, and GM-MDMs, each derived from four independent donors. Sample preparation, sequencing, and analytical methods for RNA-Seq analysis of the three LPS/IFNγ-activated MDMs were performed as previously described [18]. Following filtering criteria (≥2-fold change cut-off, *p* < 0.05), a total of 1546 transcripts were differentially expressed among M-MDM(LPS/IFNγ), GM-MDM(LPS/IFNγ), and Mob-MDM(LPS/IFNγ) (Supplementary Materials Table S1). Of the 1546 differentially expressed transcripts, 260 were identified as being upregulated in M-MDM(LPS/IFNγ) versus GM-MDM(LPS/IFNγ), while 262 were upregulated in GM-MDM(LPS/IFNγ) versus M-MDM(LPS/IFNγ). Several M1-Mφ-related genes belonging to different categories (e.g., *IL6*, *IL12B* (encoding for IL-12/23p40), *IL23A* (encoding for IL-23p19), and *IL8* (encoding for CXCL8)) were expressed at significantly higher levels in GM-MDM(LPS/IFNγ) as compared to M-MDM(LPS/IFNγ) (Table 1). On the other hand, the expression levels of a set of M2-Mφ-associated genes (e.g., *ADAMDEC1, CCL17, CD36*, *CD163*, *IL10*, *MAOA*, *SEPP1*, *SOCS2*, and *STAB1*) were significantly higher in M-MDM(LPS/IFNγ) relative to GM-MDM(LPS/IFNγ) (Table 1). Accordingly, our results clearly indicate that GM-MDM(LPS/IFNγ) and M-MDM(LPS/IFNγ) display M1-like and M2-like Mφ properties, respectively.

**Figure 2:**
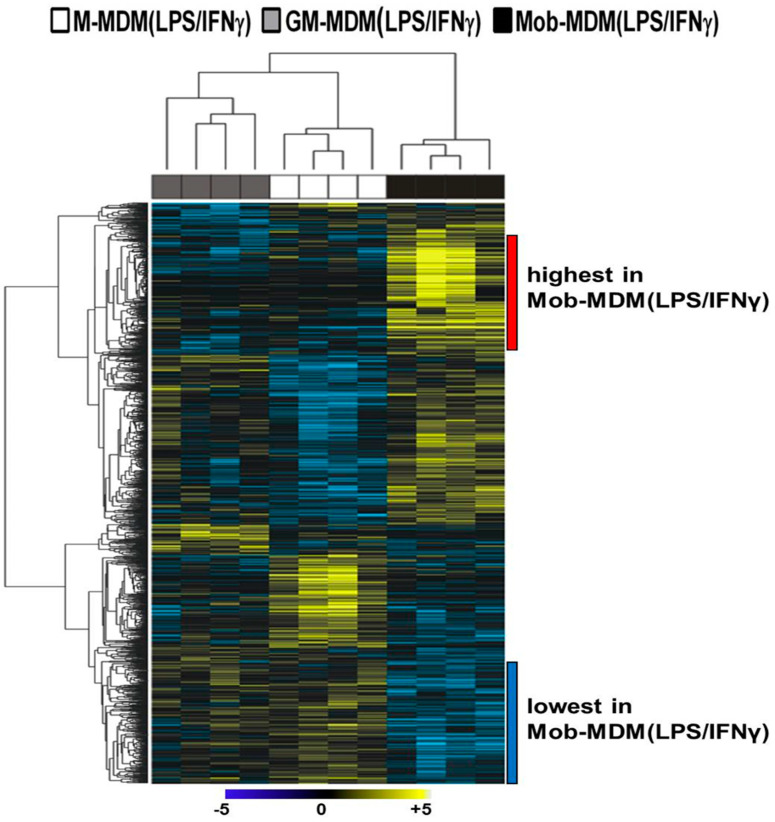
Whole-transcriptome analysis of lipopolysaccharide (LPS)/interferon γ (IFNγ)-activated monocyte-derived macrophages (MDMs). Macrophage colony-stimulating factor MDMs (M-MDMs), granulocyte/macrophage colony-stimulating factor MDMs (GM-MDMs), and heat-killed *Mycobacterium obuense* MDMs (Mob-MDMs) were generated and activated with LPS and IFNγ for 24 h as described below (see the Materials and Methods section (Section 4)) from four healthy donors (*n* = 12). Total RNA was isolated and then sequenced using the Ion Torrent platform (as described in the Materials and Methods section (Section 4)). Sequence alignment, followed by quantification of transcriptomes, was performed (as described in the Materials and Methods section (Section 4)). Transcripts (*n* = 1546) differentially expressed between M-MDM(LPS/IFNγ), GM-MDM(LPS/IFNγ), and Mob-MDM(LPS/IFNγ) were identified based on a fixed-effects model with ANOVA and analyzed by hierarchical clustering. Columns represent samples and rows constitute the differentially expressed transcripts (yellow, relatively upregulated; blue, relatively downregulated).

Hierarchical clustering analysis was performed to characterize gene clusters with differential patterns of expression among the three LPS/IFNγ-activated MDMs. A two-dimensional hierarchical clustering of both transcripts and samples demonstrated that Mob-MDM(LPS/IFNγ) formed a separate cluster from both M-MDM(LPS/IFNγ) and GM-MDM(LPS/IFNγ) (Figure 2). We noted two gene clusters that displayed the lowest and highest expression in Mob-MDM(LPS/IFNγ) relative to both M-MDM(LPS/IFNγ) and GM-MDM(LPS/IFNγ) (Figure 2). These two clusters comprised 417 transcripts that were differentially expressed in Mob-MDM(LPS/IFNγ) versus both M-MDM(LPS/IFNγ) and GM-MDM(LPS/IFNγ), but not between the latter two MDM types (Supplementary Materials Table S2). Gene ontology analysis revealed that Mob-MDM(LPS/IFNγ) profiles included cytokines/chemokines (e.g., *CSF2*, *CXCL1*, *CXCL2*, CXCL3, *CXCL5*, *CXCL9*, *CXCL10*, *CXCL11*, *IL1B*, and *IL36G*), G-protein-coupled receptors (e.g., *CXCR4* and *CXCR6*), enzymes such as peptidases, phosphatases, and kinases (e.g., *PRKCQ* and *CSNK1G3*), transmembrane receptors (e.g., *IL2RB* and *SEMA4D*), transcription regulators (e.g., *ANKRD22* and *ASB2*), and others such as *SIGLEC10* (Supplementary Materials Table S2). 

**Figure 3:**
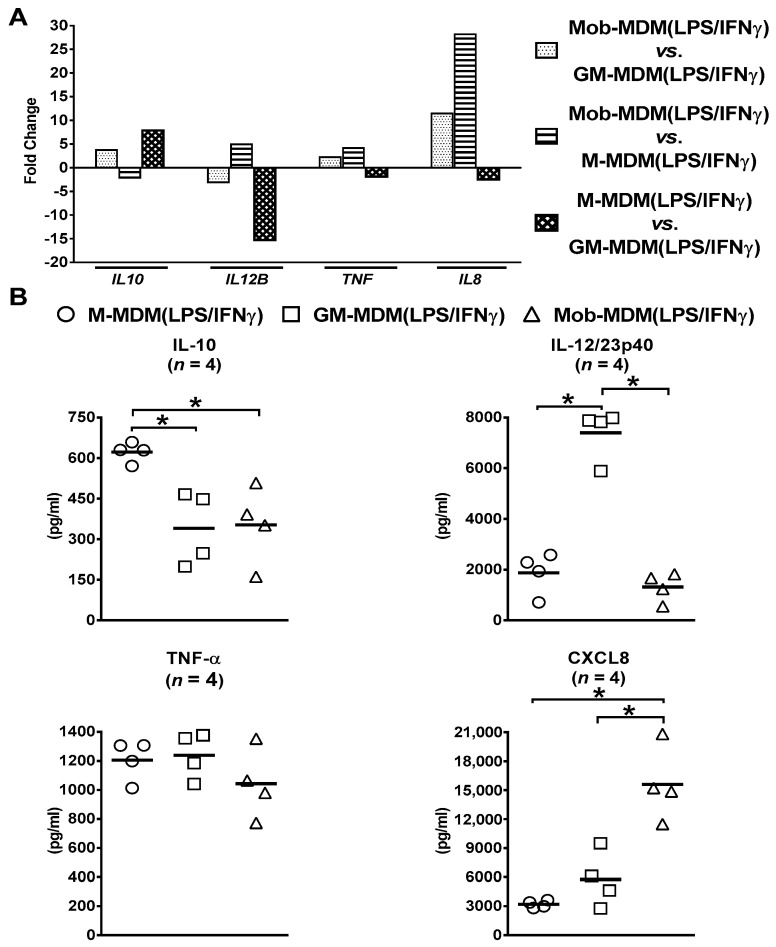
Confirmation of select cytokines and chemokines identified to be differentially expressed among lipopolysaccharide (LPS)/interferon γ (IFNγ)-activated monocyte-derived macrophages (MDMs) by RNA sequencing (RNA-Seq). Macrophage colony-stimulating factor MDMs (M-MDMs), granulocyte/macrophage colony-stimulating factor MDMs (GM-MDMs), and heat-killed *Mycobacterium obuense* MDMs (MobMDMs) were generated and were activated with LPS/IFNγ for 24 h (as described in the Materials and Methods section (Section 4)). The differential expression of (**A**) select cytokine/chemokine transcripts, namely interleukin 10 (*IL10*) (encodes IL-10), *IL12B* (encodes IL-12/23p40), tumor necrosis factor (*TNF*) (encodes TNF-α), and *IL8* (encodes chemokine C-x-C motif ligand 8 (CXCL8)), in LPS/IFNγ-activated MDMs was confirmed at (**B**) the protein level by analysis of their secreted levels in M-MDM(LPS/IFNγ), GM-MDM(LPS/IFNγ), and Mob-MDM(LPS/IFNγ) culture supernatants by ELISA. (**A**) Column bars represent fold change for each cytokine or chemokine transcript, and (**B**) scatter plots demonstrate cytokine or chemokine concentration in culture supernatants of the three LPS/IFNγ-activated MDM types. The same MDMs were used for RNA-Seq and ELISA experiments. Horizontal bars indicate group mean values of cytokine or chemokine concentration of at least 4 independent donors. One-way ANOVA, followed by Tukey’s post-hoc test, was performed to determine statistically significant differences in cytokine/chemokine secreted levels (* *p* < 0.05). Circles represent M-MDM(LPS/IFNγ), squares represent GM-MDM(LPS/IFNγ) and triangles represent Mob-MDM(LPS/IFNγ).

Orthogonal validation of RNA-Seq data found similar differential patterns of expression of select cytokines/chemokines, namely *IL8* (encodes for CXCL8), *IL10* (encodes for IL-10), *IL12B* (encodes for IL-12/23p40), and *TNF* (encodes for TNF-α), at both the transcript (Figure 3A) and protein (Figure 3B) levels in M-MDM(LPS/IFNγ), GM-MDM(LPS/IFNγ), and Mob-MDM(LPS/IFNγ).

Canonical pathway enrichment analysis identified several predicted signaling pathways that were significantly altered in Mob-MDM(LPS/IFNγ) relative to both M-MDM(LPS/IFNγ) and GM-MDM(LPS/IFNγ), but not between the latter two MDM types. These comprised, among others, activated NF-κB signaling, TREM1 signaling, and repressed p53 signaling (Table 2). 

We then performed topological network analysis to predict the activation or inhibition of potential upstream regulators of the detected set of differentially expressed genes unique to Mob-MDM(LPS/IFNγ). A total of 186 upstream regulators (121 activated (z-score ≥ 2) and 65 inhibited (z-score ≤ −2)) were identified in Mob-MDM(LPS/IFNγ) relative to M-MDM(LPS/IFNγ) and GM-MDM(LPS/IFNγ), but not between the latter MDM types (Supplementary Materials Table S3). Those predicted upstream regulators comprised various cytokines/chemokines, chemical drugs, transmembrane receptors, bacterial components, and others. CSF2 (GM-CSF; z-score = 4.61) and the LY294002 (z-score = −4.31) were predicted as the top activated and inhibited upstream regulators, respectively (Supplementary Materials Table S3). In addition, gene-set and network analysis identified biological functions (*n* = 100) significantly affected by the uniquely differentially expressed transcripts in Mob-MDM(LPS/IFNγ) compared to M-MDM(LPS/IFNγ) and GM-MDM(LPS/IFNγ), but not between the latter two MDM types (Supplementary Materials Table S4). The identified biological functions predicted to be uniquely activated (z-score ≥ 2) in Mob-MDM(LPS/IFNγ) included, among others, activation of blood cells/leukocytes, migration/cell movement of myeloid cells and Mφs, and synthesis of reactive oxygen species (Supplementary Materials Table S4). Collectively, RNA-Seq analysis revealed a distinct transcriptome profile of Mob-MDM(LPS/IFNγ) from both M-MDM(LPS/IFNγ) and GM-MDM(LPS/IFNγ).

## 3. Discussion

Mφ activation is a multifaceted and dynamic mechanism that can generate various Mφ states with a diverse range of functional outcomes [21,22]. However, the preactivation process whereby monocytes are differentiated into Mφs plays a dominant role in shaping the final activation state of Mφs irrespective of the adopted stimulus. Traditionally, GM-CSF and M-CSF have been widely used to induce monocyte-to-Mφ differentiation and to prime Mφs toward the nonactivated pre-M1 (GM-MDM) and pre-M2 (M-MDM) Mφ phenotypes, respectively [23]. Evidence from previous studies has confirmed the immunomodulatory properties and immunotherapeutic potentials of HK whole-cell mycobacteria namely, *M. vaccae* and *M. obuense*. More importantly, the safety of both HK mycobacteria is proven and well documented in several studies whereby mild adverse events were noted following the administration of either preparation to cancer [12,13,24] and human immunodeficiency virus (HIV) [25] patients. Thus, HK whole-cell preparations of *M. vaccae* and *M. obuense* have a major advantage over Bacillus Calmette–Guérin (BCG) in terms of their favorable safety and tolerability. Despite its successful usage in the treatment of nonmuscle/noninvasive bladder cancer, BCG might cause in certain cases severe and life-threatening adverse events such as local or systemic infections with BCG [26]. It has been well documented from earlier studies that various viable and killed mycobacterial species can alter the activation state of human Mφs [27,28]. In this manner, data from our previous phenotypic, functional, and transcriptomic studies have indicated that HK *M. obuense*, an immunomodulator of both the innate and adaptive immune responses, promotes the differentiation of human monocytes into a nonactivated Mφ type (Mob-MDM) that acquire proinflammatory properties suggestive of an M1-like Mφ phenotype [18]. In this study, we aimed to gain deeper insights into the cytokine/chemokine secretion patterns and transcriptome profiles of Mob-MDMs, M-MDMs, and GM-MDMs following their LPS/IFNγ-mediated activation.

In the present work and consistent with previous reports, GM-MDM(LPS/IFNγ) produced significantly higher levels of CCL22 [7,29], IL-6 [29,30], IL-12/23p40 [7,29,31], and IL-23p19/p40 [19] and significantly lower levels of IL-10 [29,30,31,32] as compared to M-MDM(LPS/IFNγ). On the other hand, a study by Vogel et al. did not find any significant difference in IL-10 secretion between M-MDM(LPS/IFNγ) and GM-MDM(LPS/IFNγ) [8]. Moreover, in sharp contrast to Verreck et al., who reported the absence of detectable levels of IL-12/23p40 in M-MDM(LPS/IFNγ) culture supernatants [19], findings from this study and from previous studies [7,31] have found M-MDM(LPS/IFNγ) to release detectable amounts of IL-12/23p40, although, to a significantly lower level than that released by GM-MDM(LPS/IFNγ). Additionally, previous studies have reported that IL-12p70 [7,32] and TNF-α [30,32] were secreted at significantly higher levels by GM-MDM(LPS/IFNγ) relative to M-MDM(LPS/IFNγ). This study only showed an overall trend, but with no statistical significance, toward increased secretion of both cytokines by GM-MDM(LPS/IFNγ) relative to M-MDM(LPS/IFNγ). Contrary to previous studies [29,32], which found CCL5 to be secreted at significantly higher levels by GM-MDM(LPS/IFNγ) relative to M-MDM(LPS/IFNγ), results from the current study demonstrated the opposite. Hence, the above-mentioned variations in the differential secretion of cytokines and chemokines between M-MDM(LPS/IFNγ) and GM-MDM(LPS/IFNγ) could be related to differences in the activation method employed by different studies, more specifically the period of exposure to LPS and IFNγ and the concentration of each activating stimulus. The profile and level of cytokines/chemokines secreted by Mφs have been widely adopted as signature markers to discriminate between the two activated M1- and M2-Mφ phenotypes [33]. Classically activated human M1-Mφs are typically characterized by their IL-10^low^, IL-12/23p40^high^ and/or IL-12p70^high^, and IL-23p19/p40^high^ phenotype [1,34]. In contrast, alternatively activated M2-Mφs have an IL-10^high^, IL-12/23p40^low^ and/or IL-12p70^low^, and IL-23p19/p40^low^ phenotype [35]. Several reports utilizing CSF-differentiated human MDMs as in vitro models to study Mφ have indicated that M-CSF and GM-CSF are crucial determinants for shaping the functional profiles of activated Mφs [29,36]. In support of this notion, this study has shown that M-MDM(LPS/IFNγ) display a cytokine secretion profile (IL-10^high^, IL-12/23p40^low^, IL-12p70^low^, and IL23p19/p40^neg^) characteristic of the M2-Mφ phenotype, while GM-MDM(LPS/IFNγ) display a cytokine secretion profile (IL-10^low^, IL-12/23p40^high^, IL-12p70^high^, and IL23p19/p40^high^) characteristic of the M1-Mφ phenotype. These striking differences were also noted at the transcript level whereby RNA-Seq analysis performed in this study revealed higher expression of *IL10* and lower expression of *IL12B* (encodes for IL-12/23p40) and *IL23A* (encodes for IL-23p19) in M-MDM(LPS/IFNγ) as compared to GM-MDM(LPS/IFNγ). This is in agreement with previous studies where similar patterns of differential mRNA expression between M-MDM(LPS/IFNγ) and GM-MDM(LPS/IFNγ) were reported for *IL10*, *IL23A* [36], and *IL12B* [31]. Therefore, despite being activated with the M1-activating stimuli, LPS and IFNγ, M-MDMs remain trapped in an M2-Mφ response mode imposed by M-CSF during the stage of monocyte-to-Mφ differentiation, and such a mode seems to be quite independent of the nature of the subsequent activating stimuli. A key finding in the current study was the demonstration that Mob-MDM(LPS/IFNγ) exhibit a distinct cytokine/chemokine secretion profile from that of M-MDM(LPS/IFNγ) and GM-MDM(LPS/IFNγ). The unique cytokine secretion profile of Mob-MDM(LPS/IFNγ) (IL-10^low^, IL-12/23p40^low^, IL-12p70^neg^, and IL23p19/p40^low^) did not correlate either with that of M1-Mφs (IL-12^high^) or with any of those of the M2-Mφ subtypes, M2a, M2b, M2c, or M2d, whereby high IL-10 production is a common feature among the four of them [37]. Moreover, the distinctiveness of Mob-MDM(LPS/IFNγ) phenotype was further reinforced by their pattern of low CXCL9 production as compared to both M-MDM(LPS/IFNγ) and GM-MDM(LPS/IFNγ). A previous study demonstrated that cell wall extracts from HK *M. obuense* and *M. vaccae* can induce the production of IL-12 and TNF-α by human THP-1-derived macrophages. In this study, IL-12 levels induced by cell wall extract of either of the two HK mycobacteria were comparable to those induced by BCG, while TNF-α levels induced by HK *M. vaccae* cell wall extract were significantly higher than those induced by BCG [38]. 

In the current study, RNA-Seq analysis was carried out to compare and contrast the transcriptome profiles of the three LPS/IFNγ-activated MDM types. RNA-Seq analysis identified 1546 transcripts that were significantly differentially expressed among M-MDM(LPS/IFNγ), GM-MDM(LPS/IFNγ), and Mob-MDM(LPS/IFNγ). A total of 417 transcripts were found to be selectively modulated in Mob-MDM(LPS/IFNγ), but not altered between M-MDM(LPS/IFNγ) and GM-MDM(LPS/IFNγ) (Supplementary Materials Table S2). Mob-MDM(LPS/IFNγ) selective transcripts encompassed a specific group of upregulated proinflammatory chemokines, namely *CXCL1*, *CXCL2*, *CXCL3*, and *CXCL5*. These four chemokines are known to share a common chemokine binding receptor, chemokine (C-X-C) motif receptor 2 (CXCR2), and they serve as potent chemoattractants for neutrophils [33,39]. On the other hand, a different family of proinflammatory chemokine transcripts (*CXCL9*, *CXCL10*, and *CXL11*) was found to be uniquely downregulated in Mob-MDM(LPS/IFNγ). This family of IFN-inducible chemokines exhibits effective T lymphocyte chemoattractant activities and is capable of binding to the cognate chemokine receptor, CXCR3 [40,41]. Moreover, other signature proinflammatory cytokines (e.g., *IFNG*, *IL1B*, and *IL36G*) were significantly upregulated in Mob-MDM(LPS/IFNγ) versus both M-MDM(LPS/IFNγ) and GM-MDM(LPS/IFNγ). Several studies have highlighted the contribution of IL-36γ (encoded by *IL-36G*) in polarizing immune cells toward type-1 immune responses, which are pertinent to antitumor immunity [42,43]. It has been previously indicated that human IL-36γ expression is inversely associated with lung and melanoma cancer progression [44]. Transcriptomic data analysis of various immune cell populations purified from human colorectal cancer (CRC) microenvironment has shown that *IL36G* is expressed at significantly higher levels in M1-Mφs compared to all other immune cells, including M2-Mφs [45,46]. Further exploration of the tumor microenvironment has uncovered a correlation between the pattern of Mφ IL-36γ expression and two positive prognostic markers for CRC which comprise CD4^+^ central memory T-cell infiltration and augmented density of B cells in tertiary lymphoid structures [46]. IL-36γ has been also reported to confer protection against severe influenza infection through enhancing the survival of mouse lung alveolar Mφs and restraining the replication of the virus [47]. Alveolar Mφs isolated from uninfected IL-36γ knockout mice exhibited an M2-like Mφ phenotype and demonstrated an increased and rapid rate of apoptosis post influenza infection [47]. Taking into account that HK *M. obuense* is currently being assessed in an ongoing clinical trial (NCT04442048) for its capacity to decrease the occurrence and severity of coronavirus disease 2019 (COVID-19)-related symptoms in cancer patients, it is noteworthy to further investigate whether there is a correlation of serum IL-36γ levels with the clinical outcomes in those patients. Results of our study identified a group of solute carriers that were selectively modulated in Mob-MDM(LPS/IFNγ), whereby *SLC27A2*, *SLC7A11*, and *SLC51B* displayed an increased expression, while *SLC8A1*, *SLC2A8*, *SLC2A5*, and *SLC6A9* showed a decreased expression. Key physiological functions for the solute carrier family include: transport of ions, uptake of nutrients, and elimination of cellular waste [48]. However, a limited number of studies have addressed the functions of the above-modulated solute carriers in human Mφs. For instance, heat-inactivated *M. tuberculosis* lysate was found to induce *SLC7A11* expression in U937-derived human Mφs [49], while *SLC8A1* mediated the release of the proinflammatory cytokine, TNFα, by human lung Mφs [50]. *SIGLEC10* was among the top 10 transcripts of its category (others) that were uniquely and significantly downregulated in Mob-MDM(LPS/IFNγ). The interaction between sialic acid-binding Ig-like lectin 10 (Siglec-10) on the surface of Mφs with CD24, commonly overexpressed on numerous human cancers, has been previously described to be involved in the suppression of Mφ-mediated immune responses to cancer [51]. Targeting the CD24-Siglec-10 signaling axis stands as a promising approach for cancer immunotherapy whereby blocking Siglec-10 on human MDMs resulted in a significant increase in the phagocytic capacity of tumor cells [52]. In this context, it would be of interest to evaluate the cytotoxic activity of Mob-MDM(LPS/IFNγ) in vitro against CD24-expressing human cancer cell lines as well as in vivo in humanized mouse cancer models.

RNA-Seq profiling coupled with pathways network analysis predicted nuclear factor-κB (NF-κB) and triggering receptor expressed on myeloid cells 1 (TREM1) signaling pathways to be significantly activated in Mob-MDM(LPS/IFNγ) relative to both M-MDM(LPS/IFNγ) and GM-MDM(LPS/IFNγ). The transcription factor, NF-κB, is a master regulator of inflammatory gene expression in Mφs [53], and it also plays a crucial role in maintaining Mφ viability [54]. It would be reasonable to expect that activation of the NF-κB signaling pathway in Mφs would induce the release of proinflammatory mediators (e.g., IL-6, inducible nitric oxide synthase (iNOs), and TNF-α) [55], hence promoting the M1-Mφ phenotype as previously indicated [56,57]. Of note, TREM1 has been previously reported to be an inducer of the M1-Mφ phenotype [58] and to extend the survival of inflammatory Mφs [59]. TREM1 activation was shown to initiate a cascade of downstream signaling events that would lead to the secretion of various M1 proinflammatory cytokines [58]. Upstream regulator analysis identified colony-stimulating factor 2 (CSF2; also known as GM-CSF) as the top activated upstream regulator in Mob-(LPS/IFNγ) compared with both M-MDM(LPS/IFNγ) and GM-MDM(LPS/IFNγ). GM-CSF has been previously reported to play a pivotal role in controlling *M. tuberculosis* infection in human MDMs in vitro, whereby the degree of infection control was positively correlated with GM-CSF secreted levels and with activated GM-CSF signaling pathways in infected Mφs [60,61]. Further studies are clearly warranted to evaluate the antimycobacterial properties of Mob-MDM(LPS/IFNγ). Taken together, the cytokine/chemokine release pattern of Mob-MDM(LPS/IFNγ), combined with their transcriptome profile, strongly point out to a unique Mφ phenotype, which is quite distinct from that of M-MDM(LPS/IFNγ) and GM-MDM(LPS/IFNγ). 

Previous work by our group indicated that nonactivated Mob-MDMs possess a potent in vitro antitumor activity whereby they displayed a higher cytostatic effect against the human pancreatic cancer cell line, BxPC3, when compared to nonactivated M-MDMs, but their effect was comparable to that of nonactivated GM-MDMs [18]. In fact, HK *M. obuense* was shown to exhibit marked immunotherapeutic effects when given in combination with chemotherapy, in patients with metastatic pancreatic cancer [13], or in combination with checkpoint inhibitors in patients with advanced melanoma [14]. In view of the aforementioned findings, it is reasonable to suggest that the antitumor activity of nonactivated or LPS/IFNγ-activated Mob-MDMs is independent of their ability to release IL-12 and might be attributed to a unique set of Mob-MDM-related genes (e.g., *CSF2* and *IL36G*). Thus, the potential association of these candidate genes with HK *M. obuense*-mediated antitumor activity needs to be pursued in future studies. More recently, there has been a strong but renewed interest in novel Mφ-based approaches in cancer immunotherapy whereby the use of chimeric antigen receptors Mφs (CAR-Ms), currently under development, has proven to be a promising immunotherapeutic tool against cancer [62,63]. In this manner, results from this study might pave the way for a potential application of HK *M. obuense* in novel cell-based immunotherapeutic modalities such as the adoptive cell transfer of autologous Mob-MDM(LPS/IFNγ) to patients with solid tumors. 

## 4. Materials and Methods

### 4.1. Blood Collection

Human whole-blood samples (150–200 mL) were obtained from anonymous phenotypically healthy adult donors through the blood bank at Nini Hospital. Whole blood was collected in plastic bags containing citrate-phosphate-dextrose-adenine and stored at room temperature for 1 h prior to being processed. 

### 4.2. Monocyte Isolation and Generation of MDMs

Whole blood was diluted with an equal volume of complete Roswell Park Memorial Institute (RPMI; Merck, Darmstadt, Germany) 1640 medium (containing 100 U/mL penicillin, 100 μg/mL streptomycin penicillin, and 2 mM L-glutamine; Merck, Darmstadt, Germany). Diluted blood was layered over Ficoll–Paque Plus (GE Healthcare, Buckingham, UK) and centrifuged at 400× *g* for 40 min at 20 °C. Peripheral blood mononuclear cells (PBMCs) were removed from the interface and washed twice with Dulbecco’s phosphate-buffered saline (DPBS; Merck, Darmstadt, Germany) by centrifugation at 200× *g* for 15 min at 20 °C to get rid of platelets. PBMCs were assessed for viability using the trypan blue exclusion method and were always >95% viable. Later, PBMCs were seeded into 75 cm^2^ tissue culture flasks at a density of 1.5 × 10^6^ cells/mL of complete RPMI medium and incubated overnight at 37 °C in a 5% CO_2_ humidified incubator. Cells were then washed thoroughly with DBPS, and adherent monocytes were permitted to differentiate for 5 days into Mφs in complete RPMI 1640 medium supplemented with 7.5% heat-inactivated pooled human AB serum (ZenBio, Durham, NC, USA) and with optimal concentrations [7,18,32] of M-CSF (100 ng/mL), GM-CSF (100 ng/mL) (R&D Systems, Abingdon, UK), or 30 µg/mL HK *M. obuense* (NCTC13365, Immodulon Therapeutics, UK) to generate M-MDMs, GM-MDMs, and Mob-MDMs, respectively. MDM purity, as assessed by flow cytometry, was ~80–85% after 5 days of differentiation.

### 4.3. Activation of MDMs

Following the 5-day monocyte-to-Mφ differentiation period, M-MDMs, GM-MDMs, and Mob-MDMs were washed thoroughly with cold DPBS and detached by gentle scraping in cold complete RPMI 1640 medium. The viability of MDMs was ~75–80%, as evaluated by the trypan blue dye exclusion method. Detached MDMs were seeded in a 24-well culture plate at a density of 3 × 10^5^ cells/well, cultured in complete RPMI 1640 medium supplemented with 10% heat-inactivated pooled human AB serum, and were activated with LPS from *Escherichia coli* O111:B4 (100 ng/mL; Merck, Darmstadt, Germany) and IFNγ (20 ng/mL; R&D Systems, Abingdon, UK) for 24 h at 37 °C in a 5% CO_2_-humidified incubator [31,32]. LPS/IFNγ-activated M-MDMs, GM-MDMs, and Mob-MDMs were referred to as M-MDM(LPS/IFNγ), GM-MDM(LPS/IFNγ), and Mob-MDM(LPS/IFNγ), respectively.

### 4.4. Measurement of Cytokine and Chemokine Levels in MDM Culture Supernatants

Supernatants of different LPS/IFNγ-activated MDM cultures were collected by centrifugation at 1000× *g* for 15 min at 4 °C, aliquoted, and stored at −80 °C for later cytokine/chemokine analysis. Levels of different cytokines (IL-6, IL-10, IL-12/23p40, IL-12p70, IL-23p19/p40, M-CSF, TGF-β1, and TNF-α) and chemokines (CCL2, CCL5, CCL22, CXCL8, and CXCL9) were measured in MDM culture supernatants using sandwich enzyme-linked immunosorbent assay (ELISA) following the protocol provided by the manufacturer (R&D Systems, Abingdon, UK). Samples were tested in duplicate, and optical densities of samples were recorded using a Multiskan Ascent microplate reader (Thermo Fisher Scientific, Waltham, MA, USA).

### 4.5. RNA Extraction from MDM

Total RNA was extracted from LPS/IFNγ-activated MDMs with the RNeasy Plus kit (Qiagen, Courtaboeuf, France) following the manufacturer’s recommendations. The RNA yield of samples was quantified using the NanoDrop 2000 spectrophotometer (Thermo Fisher Scientific, Waltham, MA, USA). RNA Quality was evaluated by calculating RNA integrity numbers (RINs) through the use of the Agilent 2100 Bioanalyzer (Agilent Technologies, Santa Clara, CA, USA) according to the manufacturer’s guidelines. RNA samples had a mean RIN of 8.9 (range = 8.7–9.2).

### 4.6. Library Preparation and RNA-Seq 

A total of 800 ng of RNA was subjected to ribosomal RNA depletion using the Low Input RiboMinus Eukaryote System v2 (Thermo Fisher Scientific, Waltham, MA, USA) following the manufacturer’s protocol. Samples were then concentrated by vacuum centrifugation, followed by the construction of whole-transcriptome libraries using the Ion Total RNA-Seq Kit v2 (Thermo Fisher Scientific, Waltham, MA, USA) according to the manufacturer’s instructions. Assessment of the size distribution of samples was carried out by using the Agilent 2100 Bioanalyzer coupled with the 6000 RNA Pico kit. Library concentrations were determined using the Agilent DNA 1000 assay on the 2100 Bioanalyzer according to the manufacturer’s procedure. Barcoded whole-transcriptome libraries were then diluted to 66 pM and pooled equally (13 μL each for a total of 26 μL) for sequencing with two samples per template preparation. All template reactions were carried out on the Ion Chef Instrument with the Ion PI Hi-Q Chef 200 kit (Thermo Fisher Scientific, Waltham, MA, USA) based on the manufacturer’s protocol. Finally, barcoded samples were loaded onto Ion PI Chips v3 (Thermo Fisher Scientific, Waltham, MA, USA), and sequencing was performed with an Ion Proton sequencer (Thermo Fisher Scientific, Waltham, MA, USA) according to the manufacturer’s instructions. 

### 4.7. RNA-Seq Data Analysis

An average of 36 million reads/sample were sequenced and attained 90% uniformity of coverage. Alignment of reads was performed using Partek flow and a two-step alignment process. Unaligned reads were initially aligned to hg19 (human reference genome) using the STAR algorithm [64]. After the initial alignment, all unaligned reads were then realigned with Bowtie2 version 2.1.0 [65], whereby reads from both alignment steps were then pooled. Transcripts were then quantified using a modified version of the expectation-maximization (E/M) algorithm, as previously defined [66]. A pseudocount was added to all values with reads per kilobase per million (RPKM) < 1.0 followed by log (base 2) transformation and quantile normalization. The fixed-effects model with ANOVA was employed to identify transcripts significantly differentially expressed (*p* < 0.05 and 2-fold change thresholds) among M-MDM(LPS/IFNγ), GM-MDM(LPS/IFNγ), and Mob-MDM(LPS/IFNγ) (*n* = 1546 transcripts, Supplementary Materials Table S1). Analysis was carried out in the R language environment [67]. Select transcripts were also evaluated in pairwise comparisons (e.g., M-MDM(LPS/IFNγ) compared to GM-MDM(LPS/IFNγ)) using *t*-tests with random variance models. Hierarchical clustering analysis was applied to distinguish, among the differentially expressed genes, those with different patterns of expression among the three LPS/IFNγ-activated MDM types [68,69]. Functional pathways analysis, including gene-set enrichment and gene–gene network analysis, of differentially expressed transcripts was conducted using Ingenuity Pathways Analysis as previously described [70]. Raw data for M-MDM(LPS/IFNγ), GM-MDM(LPS/IFNγ), and Mob-MDM(LPS/IFNγ) will be deposited into the Gene Expression Omnibus (GEO) under the dataset series GSE102492.

### 4.8. Statistical Analysis

Statistical analysis was carried out using GraphPad Prism software (version 6; GraphPad Inc., San Diego, CA, USAe). Cytokine/chemokine data are presented as mean values ± standard error of the mean (SEM). One-way ANOVA, followed by Tukey’s multiple comparison post-hoc test, was used to analyze cytokine/chemokine data. Values of *p* < 0.05 denoted the existence of a statistically significant difference between compared groups.

## Figures and Tables

**Table 1:** Select transcripts significantly differentially expressed between lipopolysaccharide (LPS)/interferon γ (IFNγ)-activated human monocyte-derived macrophages (MDMs) that were differentiated with macrophage colony-stimulating factor (M-MDM(LPS/IFNγ)) and granulocyte/macrophage colony-stimulating factor (GM-MDM(LPS/IFNγ)).

RefSeq Transcript ID	Gene Symbol	Entrez Gene Name	FC (vs. GM-MDM(LPS/IFNγ))
NM_002933	*RNASE1*	Ribonuclease, RNase A family, 1	20.65
NM_006274	*CCL19*	Chemokine (C-C motif) ligand 19	18.02
NM_001400	*S1PR1*	sphingosine-1-phosphate receptor 1	11.92
NM_000240	*MAOA*	monoamine oxidase A	10.01
NM_000572	*IL10*	Interleukin 10	7.88
NM_203416	*CD163*	CD163 molecule	7.44
NM_004244	*CD163*	CD163 molecule	7.38
NM_015136	*STAB1*	Stabilin 1	6.72
NM_001270471	*SOCS2*	Suppressor of cytokine signaling 2	5.16
NM_001270467	*SOCS2*	Suppressor of cytokine signaling 2	3.97
NM_014479	*ADAMDEC1*	ADAM-like, decysin 1	3.64
NM_001127443	*CD36*	CD36 molecule	3.55
NM_002987	*CCL17*	Chemokine (C-C motif) ligand 17	3.45
NM_003877	*SOCS2*	Suppressor of cytokine signaling 2	3.22
NM_004994	*MMP9*	Matrix metallopeptidase 9	2.82
NM_001127444	*CD36*	CD36 molecule	2.82
NM_001001547	*CD36*	CD36 molecule	2.63
NM_001001548	*CD36*	CD36 molecule	2.62
NM_181054	*HIF1A*	Hypoxia-inducible factor 1, alpha subunit (basic helix–loop–helix transcription factor)	2.48
NM_000072	*CD36*	CD36 molecule	2.29
NM_005410	*SEPP1*	Selenoprotein P, plasma, 1	2.13
NM_016584	*IL23A*	Interleukin 23 subunit alpha (p19)	−2.34
NM_000584	*IL8*	Chemokine (C-X-C motif) ligand 8	−2.46
NM_000759	*CSF3*	Colony-stimulating factor 3	−2.49
NM_003775	*S1PR4*	Sphingosine-1-phosphate receptor 4	−2.64
NM_001764	*CD1B*	CD1b molecule	−3.39
NM_000575	*IL1A*	Interleukin 1 alpha	−3.63
NM_002438.1	*MRC1*	Mannose receptor, C type 1 (CD206)	−3.96
NM_001025194	*CES1*	Carboxylesterase 1	−3.97
NM_002990	*CCL22*	Chemokine (C-C motif) ligand 22	−4.08
NM_001025195	*CES1*	Carboxylesterase 1	−4.22
NM_001207019	*FCER2*	Fc fragment of IgE receptor II	−4.23
NM_002438	*MRC1*	Mannose receptor, C type 1 (CD206)	−4.37
NM_001266	*CES1*	carboxylesterase 1	−4.38
NM_000600	*IL6*	Interleukin 6	−4.63
NM_006770	*MARCO*	Macrophage receptor with collagenous structure	−7.49
NM_002981	*CCL1*	Chemokine (C-C motif) ligand 1	−8.71
NM_002187	*IL12B*	Interleukin 12B (p40)	−15.28

FC: fold change.

**Table 2:** Signaling pathways that are uniquely modulated in lipopolysaccharide (LPS)/interferon γ (IFNγ)-activated human monocyte-derived macrophages (MDMs) that were differentiated with HK *M. obuense* (Mob-MDM(LPS/IFNγ)) relative to those differentiated with macrophage colony-stimulating factor (M-MDM(LPS/IFNγ)) and granulocyte/macrophage colony-stimulating factor (GM-MDM(LPS/IFNγ)).

	Activation Z-Score	
Signaling Pathway	Mob-MDM(LPS/IFNγ) *vs.* GM-MDM(LPS/IFNγ) and M-MDM(LPS/IFNγ)	M-MDM(LPS/IFNγ) *vs*. GM-MDM(LPS/IFNγ)
Endothelin-1 Signaling	2.65	−0.38
NF-κB Signaling	2.65	−0.82
Estrogen-mediated S-phase Entry	2.45	1.63
Mitotic Roles of Polo-Like Kinase	2.45	0.82
Role of IL-17F in Allergic Inflammatory Airway Diseases	2.24	0.45
TREM1 Signaling	2.24	−0.45
p53 Signaling	−2.65	1.13

## Data Availability

Raw data for M-MDM(LPS/IFNγ), GM-MDM(LPS/IFNγ), and Mob-MDM(LPS/IFNγ) will be deposited into the Gene Expression Omnibus (GEO) under the dataset series.

## Supplementary Materials

The following are available online at https://www.mdpi.com/article/10.3390/ijms22137214/s1.
