# Supplementary material for: Cytokine/Chemokine Release Patterns and Transcriptomic Profiles of LPS/IFNγ-Activated Human Macrophages Differentiated with Heat-Killed Mycobacterium obuense, M-CSF, or GM-CSF
Source: Int J Mol Sci. 2021 Jul 5;22(13):7214. doi: 10.3390/ijms22137214 (PMC8268300; doi:10.3390/ijms22137214)
Supplement: Supplementary file 1 [file ijms-22-07214-s001.zip › ijms-1267707-supplementary.pdf]

Table S1. Transcripts that are significantly differentially expressed among M-MDM(LPS/IFNγ), GM-MDM(LPS/IFNγ), and Mob-MDM(LPS/IFNγ).

		Fold Change		
**RefSeq transcript ID**	**Symbol**	**Mob-MDM(LPS/IFNγ) *vs.* GM-MDM(LPS/IFNγ)**	**Mob-MDM(LPS/IFNγ) *vs.* M-MDM(LPS/IFNγ)**	**M-MDM(LPS/IFNγ) *vs.* GM-MDM(LPS/IFNγ)**
NM_000758	*CSF2*	38.14	47.90	-1.26
NM_000619	*IFNG*	34.43	22.54	1.53
NM_002994	*CXCL5*	27.00	18.59	1.45
NM_004131	*GZMB*	26.41	12.47	2.12
NM_000096	*CP*	21.10	19.66	1.07
NM_000878	*IL2RB*	19.00	8.68	2.19
NM_014767	*SPOCK2*	18.48	8.22	2.25
NM_016584	*IL23A*	18.25	42.79	-2.34
NM_172219	*CSF3*	17.84	16.66	1.07
NM_003537	*HIST1H3B*	16.97	14.05	1.21
NM_003531	*HIST1H3C*	16.43	11.00	1.49
NM_003534	*HIST1H3G*	15.88	11.89	1.34
NM_001767	*CD2*	14.85	11.00	1.35
NM_005322	*HIST1H1B*	13.48	6.97	1.93
NM_001005464.1	*HIST2H3A*	13.48	8.08	1.67
NM_021059.1	*HIST2H3C*	13.48	8.08	1.67
NM_001034	*RRM2*	12.53	13.10	-1.05
NM_021066	*HIST1H2AJ*	12.50	8.81	1.42
NR_046035	*CXCL1*	12.45	7.96	1.56
NM_003521	*HIST1H2BM*	12.44	9.80	1.27
NM_000584	*IL8*	11.47	28.21	-2.46
NM_003511	*HIST1H2AL*	10.45	6.26	1.67
NR_033662	*CSF3*	10.17	13.61	-1.34
NM_021018	*HIST1H3F*	10.00	6.18	1.62
NR_046371	*CP*	9.91	7.26	1.36
NM_019618	*IL36G*	9.73	10.75	-1.10
NM_000576	*IL1B*	9.52	10.43	-1.09
NM_001144966	*NEDD4L*	9.46	10.34	-1.09
NM_003535	*HIST1H3J*	8.74	7.73	1.13
NM_001071	*TYMS*	8.53	5.47	1.56
NM_002561	*P2RX5*	8.27	10.28	-1.24
NM_001161443	*SH2D2A*	8.23	6.96	1.18
NM_000733	*CD3E*	8.18	7.71	1.06

NM_030915	*LBH*	8.14	5.32	1.53
NM_005356	*LCK*	7.92	7.92	1.00
NM_003519	*HIST1H2BL*	7.88	3.87	2.04
NM_024508	*ZBED2*	7.81	5.77	1.35
NM_052916	*RNF157*	7.76	7.44	1.04
NM_001005464	*HIST2H3A*	7.70	7.51	1.03
NM_021059	*HIST2H3C*	7.70	7.51	1.03
NM_001083116	*PRF1*	7.69	5.24	1.47
NM_001145966	*MKI67*	7.50	5.53	1.36
NM_052838	*1-Sep*	7.35	5.06	1.45
NM_001511	*CXCL1*	7.34	8.90	-1.21
NM_005442	*EOMES*	7.25	4.92	1.48
NM_198827	*GPR133*	7.25	15.87	-2.19
NM_002466	*MYBL2*	7.19	6.33	1.13
NM_003509	*HIST1H2AI*	7.10	3.43	2.07
NM_012092	*ICOS*	7.04	6.73	1.04
NM_005238	*ETS1*	6.98	2.57	2.72
NM_006564	*CXCR6*	6.93	7.04	-1.02
NM_017709	*FAM46C*	6.61	6.23	1.06
NM_005816	*CD96*	6.60	4.17	1.58
NM_001042600	*MAP4K1*	6.57	5.59	1.17
NM_001130046	*CCL20*	6.48	13.12	-2.03
NM_003645	*SLC27A2*	6.39	5.92	1.08
NM_001143965	*TBC1D7*	6.32	2.72	2.32
NM_002090	*CXCL3*	6.26	3.17	1.97
NM_004195	*TNFRSF18*	6.16	9.76	-1.58
NM_000655	*SELL*	6.12	5.45	1.12
NM_006433	*GNLY*	6.12	4.85	1.26
NM_000759	*CSF3*	6.10	15.20	-2.49
NM_002526	*NT5E*	5.87	15.90	-2.71
NM_015277	*NEDD4L*	5.86	2.46	2.39
NM_175055	*HIST3H2BB*	5.85	3.76	1.56
NM_206925	*CA12*	5.83	4.34	1.34
NM_006144	*GZMA*	5.81	5.08	1.14
NM_006725	*CD6*	5.79	5.46	1.06
NM_021064	*HIST1H2AG*	5.78	2.96	1.95
NM_018410	*HJURP*	5.72	5.72	1.00
NM_003514	*HIST1H2AM*	5.70	2.87	1.99
NM_001079	*ZAP70*	5.65	5.02	1.12
NM_021062	*HIST1H2BB*	5.63	2.68	2.10

NM_004430	*EGR3*	5.59	4.98	1.12
NM_003536	*HIST1H3H*	5.58	3.07	1.82
NM_016448	*DTL*	5.55	5.52	1.01
NM_001131010	*SATB1*	5.50	4.42	1.25
NM_173157	*NR4A1*	5.40	9.23	-1.71
NM_004591	*CCL20*	5.38	12.77	-2.37
NM_001007466	*TULP4*	5.33	1.81	2.95
NM_003548	*HIST2H4A*	5.32	3.15	1.69
NM_001034077	*HIST2H4B*	5.32	3.15	1.69
NM_003513	*HIST1H2AB*	5.28	3.73	1.42
NM_007288	*MME*	5.26	2.33	2.26
NM_001144970	*NEDD4L*	5.25	3.34	1.57
NM_014220	*TM4SF1*	5.24	4.60	1.14
NM_015482	*SLC22A23*	5.18	-1.53	7.91
NM_033423	*GZMH*	5.16	3.47	1.49
NM_003527	*HIST1H2BO*	5.14	3.00	1.71
NM_001270472	*MCM3*	5.13	2.46	2.09
NM_018136	*ASPM*	5.07	5.85	-1.15
NM_001718	*BMP6*	5.06	2.34	2.16
NM_006257	*PRKCQ*	5.04	3.62	1.39
NM_001123375	*HIST2H3D*	5.02	3.18	1.58
NM_001559	*IL12RB2*	5.00	5.00	1.00
NM_080596	*HIST1H2AH*	4.94	2.13	2.31
NM_003853	*IL18RAP*	4.92	4.44	1.11
NM_000575	*IL1A*	4.92	17.83	-3.63
NM_133272	*FCAR*	4.87	4.42	1.10
NM_178844	*NLRC3*	4.86	3.69	1.32
NM_004091	*E2F2*	4.86	4.72	1.03
NM_001243121	*PDE4A*	4.82	2.97	1.62
NM_001067	*TOP2A*	4.81	5.10	-1.06
NM_001394	*DUSP4*	4.77	9.18	-1.92
NM_002000	*FCAR*	4.77	4.41	1.08
NM_018004	*TMEM45A*	4.71	3.23	1.46
NR_027353	*CD8A*	4.67	2.12	2.20
NM_003533	*HIST1H3I*	4.66	2.32	2.01
NM_006137	*CD7*	4.65	3.05	1.52
NM_001218	*CA12*	4.59	4.68	-1.02
NM_000734	*CD247*	4.56	5.37	-1.18
NM_002263.1	*KIFC1*	4.55	4.61	-1.01
NM_012223	*MYO1B*	4.53	6.32	-1.39

NM_002964	*S100A8*	4.53	2.12	2.14
NM_002089	*CXCL2*	4.51	3.87	1.16
NM_003548.1	*HIST2H4A*	4.50	2.84	1.59
NM_001034077.1	*HIST2H4B*	4.50	2.84	1.59
NM_001267058	*CASP7*	4.47	1.86	2.40
NM_201538	*NDRG2*	4.47	4.47	1.00
NM_145019	*FAM124A*	4.45	7.92	-1.78
NM_016343	*CENPF*	4.42	3.12	1.42
NM_021058	*HIST1H2BJ*	4.39	2.35	1.87
NM_001168	*BIRC5*	4.37	4.30	1.02
NM_003546	*HIST1H4L*	4.36	3.42	1.28
NM_001164114	*CASS4*	4.36	4.68	-1.07
NM_032415	*CARD11*	4.32	4.84	-1.12
NM_002575	*SERPINB2*	4.30	4.57	-1.06
NM_031445	*AMMECR1L*	4.29	-1.05	4.48
NM_002178	*IGFBP6*	4.25	1.91	2.23
NM_003539	*HIST1H4D*	4.24	2.88	1.47
NM_000732	*CD3D*	4.20	4.20	1.00
NM_012112	*TPX2*	4.19	4.78	-1.14
NM_002775	*HTRA1*	4.18	1.82	2.30
NM_013308	*GPR171*	4.15	3.84	1.08
NM_003495	*HIST1H4I*	4.15	1.83	2.26
NM_001164586	*IGFN1*	4.11	13.13	-3.19
NM_001008540	*CXCR4*	4.06	3.49	1.16
NM_003538	*HIST1H4A*	4.05	3.66	1.11
NM_006845	*KIF2C*	4.05	5.06	-1.25
NM_002928	*RGS16*	4.05	2.14	1.89
NM_001253792	*ZNF444*	4.04	1.86	2.18
NR_026692	*CD209*	4.03	6.42	-1.59
NM_020356	*CASS4*	4.02	3.41	1.18
NM_003541	*HIST1H4K*	4.01	1.83	2.19
NM_001255	*CDC20*	3.99	3.44	1.16
NM_001130158	*MYO1B*	3.99	6.22	-1.56
NM_001680	*FXYD2*	3.98	3.98	1.00
NM_201541	*NDRG2*	3.98	16.88	-4.24
NM_003523	*HIST1H2BE*	3.97	2.46	1.62
NM_000245	*MET*	3.93	5.07	-1.29
NM_002875	*RAD51*	3.92	2.97	1.32
NM_004603	*STX1A*	3.92	2.47	1.59
NM_014391	*ANKRD1*	3.92	13.56	-3.46

NM_002984	*CCL4*	3.90	2.90	1.35
NR_037716	*RNASEK*	3.88	1.97	1.97
NM_003650	*CST7*	3.86	2.01	1.92
NM_004523	*KIF11*	3.85	3.87	-1.00
NM_003510	*HIST1H2AK*	3.84	1.96	1.95
NM_175840	*SMOX*	3.83	5.21	-1.36
NM_019074	*DLL4*	3.82	-3.72	14.20
NM_001204519	*P2RX5*	3.81	2.89	1.32
NM_003529	*HIST1H3A*	3.81	1.82	2.09
NM_005225	*E2F1*	3.79	5.86	-1.55
NM_002467	*MYC*	3.78	2.84	1.33
NM_003524	*HIST1H2BH*	3.77	3.07	1.23
NM_057158	*DUSP4*	3.75	8.71	-2.32
NM_020530	*OSM*	3.75	5.81	-1.55
NM_000572	*IL10*	3.74	-2.11	7.88
NM_016426	*GTSE1*	3.72	3.72	1.00
NM_001258216	*IL12RB2*	3.72	3.21	1.16
NM_032571	*EMR3*	3.70	9.81	-2.65
NM_001003675	*LDLRAD4*	3.70	2.79	1.33
NM_080722	*ADAMTS14*	3.69	2.17	1.70
NM_139155	*ADAMTS14*	3.69	2.17	1.70
NM_005320	*HIST1H1D*	3.68	1.83	2.01
NM_018349	*MCTP2*	3.66	3.09	1.18
NM_000212	*ITGB3*	3.66	6.34	-1.73
NM_006474	*PDPN*	3.65	-1.54	5.60
NM_004336	*BUB1*	3.65	4.44	-1.22
NM_001002017	*HCFC1R1*	3.60	1.74	2.07
NM_012483	*GNLY*	3.60	2.73	1.32
NM_005733	*KIF20A*	3.56	3.44	1.04
NM_002965	*S100A9*	3.56	2.02	1.76
NM_003525	*HIST1H2BI*	3.55	2.37	1.50
NM_002609	*PDGFRB*	3.51	1.68	2.08
NM_012481	*IKZF3*	3.50	3.03	1.16
NM_001163524	*ITPRIPL1*	3.50	2.70	1.30
NM_005601	*NKG7*	3.49	1.22	2.86
NM_001165931	*RRM2*	3.48	3.48	1.00
NM_007287	*MME*	3.47	3.07	1.13
NM_182914	*SYNE2*	3.47	3.87	-1.12
NM_001992	*F2R*	3.46	3.37	1.03
NM_001136232	*SEC13*	3.46	2.50	1.38

NM_001109	*ADAM8*	3.44	1.33	2.58
NM_001202233	*NR4A1*	3.44	4.16	-1.21
NM_005916	*MCM7*	3.43	2.91	1.18
NM_152515	*CKAP2L*	3.43	3.43	1.00
NM_001145664	*RFX8*	3.43	3.43	1.00
NM_003540	*HIST1H4F*	3.41	1.84	1.86
NM_015441	*OLFML2B*	3.40	-1.73	5.89
NM_005030	*PLK1*	3.39	2.90	1.17
NM_030754	*SAA2*	3.37	2.54	1.33
NR_073375	*MCM2*	3.33	4.31	-1.29
NM_001237	*CCNA2*	3.33	2.78	1.20
NM_014207	*CD5*	3.32	2.86	1.16
NM_001785	*CDA*	3.32	10.59	-3.19
NM_001257410	*IKZF3*	3.32	2.66	1.24
NM_001271099	*PUF60*	3.28	1.71	1.91
NM_001254	*CDC6*	3.26	3.53	-1.08
NM_017720	*STAP2*	3.25	2.21	1.47
NM_001197221	*PDE4D*	3.25	3.90	-1.20
NM_024007	*EBF1*	3.25	3.01	1.08
NM_001244950	*SPOCK2*	3.25	4.77	-1.47
NM_001079907	*ZNF331*	3.25	2.61	1.24
NM_032108	*SEMA6B*	3.24	3.50	-1.08
NM_001262	*CDKN2C*	3.23	2.54	1.27
NM_014479	*ADAMDEC1*	3.21	-1.13	3.64
NM_001242312	*FAM124A*	3.21	10.73	-3.35
NM_001786	*CDK1*	3.20	2.76	1.16
NM_000600	*IL6*	3.18	14.74	-4.63
NM_018643	*TREM1*	3.18	10.27	-3.23
NM_201540	*NDRG2*	3.18	7.66	-2.41
NM_144720	*JAKMIP1*	3.16	3.16	1.00
NM_002608	*PDGFB*	3.16	4.28	-1.35
NM_003975	*SH2D2A*	3.15	3.15	1.00
NM_002417	*MKI67*	3.15	8.77	-2.79
NR_038380	*SLC7A11-AS1*	3.14	3.63	-1.15
NM_001144894	*CD209*	3.14	2.98	1.05
NM_003522	*HIST1H2BF*	3.14	2.49	1.26
NM_012258	*HEY1*	3.14	3.91	-1.25
NM_001271606	*BASP1*	3.13	1.17	2.67
NM_003544	*HIST1H4B*	3.12	2.57	1.22
NM_145687	*MAP4K4*	3.11	4.13	-1.33

NM_000639	*FASLG*	3.10	2.51	1.24
NM_198042	*PDLIM2*	3.10	5.01	-1.62
NM_004428	*EFNA1*	3.08	4.12	-1.34
NM_177963	*SYT12*	3.08	7.37	-2.39
NM_013282	*UHRF1*	3.07	2.71	1.13
NM_016441	*CRIM1*	3.06	3.12	-1.02
NM_004994	*MMP9*	3.06	1.08	2.82
NM_021968	*HIST1H4J*	3.06	1.64	1.87
NM_001099439	*EPHA10*	3.04	3.03	1.00
NM_000608	*ORM2*	3.02	3.17	-1.05
NM_000403	*GALE*	3.01	6.46	-2.14
NM_001197222	*PDE4D*	3.01	3.30	-1.10
NM_138768	*MYEOV*	3.00	4.66	-1.55
NM_001264573	*KIF18B*	3.00	2.87	1.05
NM_001145271	*ADAMDEC1*	3.00	-1.16	3.46
NM_000228	*LAMB3*	2.99	2.45	1.22
NM_022770	*GINS3*	2.99	2.85	1.05
NM_005214	*CTLA4*	2.98	6.60	-2.22
NM_001243198	*HIP1*	2.97	5.56	-1.87
NM_002082	*GRK6*	2.97	2.51	1.18
NM_002983	*CCL3*	2.97	3.31	-1.12
NM_015071	*ARHGAP26*	2.95	1.71	1.73
NM_003542	*HIST1H4C*	2.94	2.19	1.34
NM_014331	*SLC7A11*	2.91	2.67	1.09
NM_001657.1	*AREG*	2.91	2.92	-1.00
NM_014875	*KIF14*	2.90	2.90	1.00
NM_001243985	*RELA*	2.90	1.22	2.37
NM_001252102	*KIF21B*	2.89	3.15	-1.09
NM_001242607	*NCAM1*	2.88	3.81	-1.32
NR_024273	*SEC13*	2.88	2.65	1.09
NM_001257360	*AMPD2*	2.86	3.27	-1.14
NM_004431	*EPHA2*	2.85	5.64	-1.97
NM_001177428	*TRPV4*	2.85	4.41	-1.54
NM_173676	*PNPLA1*	2.85	3.96	-1.39
NM_152468	*TMC8*	2.84	3.20	-1.12
NM_001161819	*MYO1B*	2.84	13.18	-4.64
NM_001077511.1	*TCF19*	2.84	2.01	1.41
NM_033016	*PDGFB*	2.82	4.60	-1.63
NM_004130	*GYG1*	2.80	3.00	-1.07
NR_073484	*CDK2AP2*	2.79	2.25	1.24

NM_005276	*GPD1*	2.79	4.52	-1.62
NM_001006947	*UHRF1BP1L*	2.78	1.01	2.75
NM_021953	*FOXM1*	2.78	3.54	-1.28
NM_000073	*CD3G*	2.77	2.77	1.00
NM_006907	*PYCR1*	2.77	2.76	1.01
NM_021155	*CD209*	2.77	6.84	-2.47
NM_012310	*KIF4A*	2.77	2.77	1.00
NM_182802	*MCM8*	2.77	1.78	1.55
NM_001211	*BUB1B*	2.76	2.94	-1.06
NM_002236	*KCNF1*	2.76	5.49	-1.99
NM_173483	*CYP4F22*	2.75	4.84	-1.76
NM_080881	*DBN1*	2.75	2.51	1.10
NM_206918	*DEGS2*	2.73	4.24	-1.55
NM_001161560	*TNIK*	2.73	2.27	1.20
NM_174918	*C19orf59*	2.72	10.98	-4.03
NM_001001323	*ATP2B1*	2.72	2.68	1.02
NM_014400	*LYPD3*	2.72	6.31	-2.32
NM_213655	*WNK1*	2.71	1.60	1.70
NM_006186	*NR4A2*	2.71	3.16	-1.16
NM_024070	*PVRIG*	2.71	1.95	1.39
NM_005319	*HIST1H1C*	2.71	1.63	1.66
NM_003530	*HIST1H3D*	2.70	2.13	1.27
NM_003543	*HIST1H4H*	2.70	1.74	1.55
NM_017640	*LRRC16A*	2.69	2.40	1.12
NM_203394	*E2F7*	2.67	3.35	-1.25
NM_000094	*COL7A1*	2.67	3.06	-1.15
NM_178834	*LAYN*	2.66	4.25	-1.60
NM_000963	*PTGS2*	2.66	1.98	1.34
NM_001242416	*WDR20*	2.65	2.44	1.09
NM_003855	*IL18R1*	2.65	2.57	1.03
NM_003579	*RAD54L*	2.65	2.64	1.00
NM_000954	*PTGDS*	2.65	5.69	-2.15
NM_001017995	*SH3PXD2B*	2.65	2.02	1.31
NM_001789	*CDC25A*	2.63	2.61	1.01
NM_134262	*RORA*	2.63	2.69	-1.02
NM_003582	*DYRK3*	2.62	1.72	1.52
NM_001113378	*FANCI*	2.61	2.96	-1.14
NM_012168	*FBXO2*	2.60	2.60	1.00
NM_003095	*SNRPF*	2.60	1.53	1.71
NM_001042728	*RARG*	2.60	3.05	-1.17

NM_002358	*MAD2L1*	2.60	2.82	-1.08
NM_001172128	*IL16*	2.60	3.46	-1.33
NM_001400	*S1PR1*	2.59	-4.60	11.92
NM_001256511	*SSBP1*	2.59	1.79	1.45
NM_005723	*TSPAN5*	2.58	5.14	-1.99
NM_004867	*ITM2A*	2.58	2.58	1.00
NM_198517	*TBC1D10C*	2.58	2.44	1.06
NM_002122	*HLA-DQA1*	2.57	3.85	-1.49
NM_001256295	*ETS2*	2.57	1.32	1.95
NM_021777	*ADAM28*	2.57	-1.12	2.88
NM_001242608	*NCAM1*	2.57	4.47	-1.74
NM_002638	*PI3*	2.56	1.90	1.35
NM_006101	*NDC80*	2.56	2.86	-1.12
NM_001335	*CTSW*	2.55	3.53	-1.38
NM_173799	*TIGIT*	2.55	2.73	-1.07
NM_001161562	*TNIK*	2.53	2.10	1.21
NM_022898	*BCL11B*	2.52	2.30	1.10
NM_182666	*UBE2E1*	2.51	1.64	1.53
NM_207336	*ZNF467*	2.51	1.38	1.82
NM_033319	*CENPL*	2.51	1.52	1.65
NM_014965	*TRAK1*	2.51	1.26	1.99
NM_138371	*PCED1B*	2.51	2.07	1.21
NM_173091	*NFATC2*	2.51	2.51	1.00
NM_001015049	*BAG5*	2.51	1.04	2.42
NM_001256875	*CDCA8*	2.51	3.24	-1.29
NM_001198786	*POU2F1*	2.50	1.30	1.92
NM_002155	*HSPA6*	2.49	1.26	1.98
NM_002224	*ITPR3*	2.48	2.57	-1.04
NM_005239	*ETS2*	2.48	1.74	1.42
NM_138295	*PKD1L1*	2.47	3.72	-1.51
NM_013372	*GREM1*	2.47	2.41	1.02
NM_021642	*FCGR2A*	2.47	1.04	2.37
NM_001220767	*IKZF1*	2.47	1.43	1.72
NM_001267050	*GPR155*	2.46	1.67	1.48
NM_001017420	*ESCO2*	2.46	2.29	1.08
NM_178859	*SLC51B*	2.45	3.48	-1.42
NM_001017917	*CYB561*	2.45	2.34	1.04
NM_001271749	*GPR77*	2.45	2.64	-1.08
NM_004701	*CCNB2*	2.44	3.39	-1.39
NM_000615	*NCAM1*	2.44	8.00	-3.28

NM_017789	*SEMA4C*	2.44	2.18	1.12
NM_021052	*HIST1H2AE*	2.44	1.43	1.70
NM_181054	*HIF1A*	2.43	-1.02	2.48
NM_004936	*CDKN2B*	2.42	2.07	1.17
NM_145686	*MAP4K4*	2.42	4.28	-1.77
NM_012145	*DTYMK*	2.42	1.46	1.65
NM_013252	*CLEC5A*	2.42	10.94	-4.52
NM_001243283	*ALCAM*	2.42	1.77	1.36
NM_001145199	*C12orf75*	2.42	2.42	1.00
NM_001202514	*MXD1*	2.42	2.66	-1.10
NM_007350	*PHLDA1*	2.41	3.17	-1.32
NM_020689	*SLC24A3*	2.41	3.62	-1.51
NR_028308	*BRE-AS1*	2.41	2.50	-1.04
NM_080616	*C20orf112*	2.41	2.58	-1.07
NM_021615	*CHST6*	2.39	2.61	-1.09
NM_001042428	*ZNF205*	2.39	-1.32	3.16
NM_199185	*NPM1*	2.39	2.18	1.10
NM_001171992	*C6orf223*	2.38	-1.07	2.56
NM_017444	*CHRAC1*	2.38	1.48	1.60
NM_018131	*CEP55*	2.38	2.80	-1.18
NM_031966	*CCNB1*	2.38	2.87	-1.21
NM_018518	*MCM10*	2.37	2.37	1.00
NR_034033	*LOC285972*	2.37	5.08	-2.15
NM_001139500	*FGF13*	2.36	1.82	1.30
NM_175839	*SMOX*	2.36	5.60	-2.37
NM_005338	*HIP1*	2.36	4.00	-1.70
NM_004217	*AURKB*	2.36	2.54	-1.08
NM_015297	*KIAA1045*	2.36	2.71	-1.15
NM_014450	*SIT1*	2.36	2.36	1.00
NM_006096	*NDRG1*	2.35	2.26	1.04
NM_152562	*CDCA2*	2.35	4.53	-1.92
NM_182665	*RASSF5*	2.35	3.22	-1.37
NM_198947	*FAM111B*	2.35	2.35	1.00
NM_201536	*NDRG2*	2.34	2.34	1.00
NM_001131027	*PDLIM4*	2.34	7.60	-3.25
NM_001040694	*INCENP*	2.33	1.51	1.54
NM_182751	*MCM10*	2.32	2.30	1.01
NR_027767	*TNIK*	2.32	2.85	-1.23
NM_201539	*NDRG2*	2.31	2.05	1.13
NM_003467	*CXCR4*	2.31	2.06	1.12

NM_012193	*FZD4*	2.30	6.47	-2.81
NM_018154	*ASF1B*	2.29	3.89	-1.70
NM_001195728	*SLC1A2*	2.29	3.24	-1.41
NM_006591	*POLD3*	2.29	1.32	1.73
NR_002720	*TRPC2*	2.28	2.61	-1.14
NM_006693	*CPSF4*	2.28	1.09	2.09
NM_001198624	*TNFSF13*	2.28	3.04	-1.33
NR_027163	*C17orf76-AS1*	2.28	-1.14	2.58
NM_017594	*DIRAS2*	2.27	9.48	-4.18
NM_020354	*ENTPD7*	2.27	1.69	1.34
NM_001570	*IRAK2*	2.27	3.06	-1.35
NM_001002800	*SMC4*	2.26	1.95	1.16
NM_021149	*COTL1*	2.26	3.30	-1.46
NM_001206917	*CACNB3*	2.26	1.46	1.55
NM_014750	*DLGAP5*	2.25	2.25	1.00
NM_001243	*TNFRSF8*	2.25	-3.41	7.66
NM_032514	*MAP1LC3A*	2.24	-1.56	3.50
NM_017946	*FKBP14*	2.24	2.78	-1.24
NM_005508	*CCR4*	2.23	2.23	1.00
NM_001170794	*BACH2*	2.23	3.56	-1.59
NM_000594.5	*TNF*	2.23	4.19	-1.88
NM_002988	*CCL18*	2.23	1.27	1.75
NM_015845	*MBD1*	2.23	1.38	1.62
NM_005252	*FOS*	2.22	3.59	-1.61
NM_001159531	*BEGAIN*	2.22	2.06	1.08
NM_001199797	*PTPN7*	2.22	2.99	-1.35
NR_028076	*SCARF1*	2.22	2.17	1.02
NM_022756	*MEAF6*	2.21	2.65	-1.20
NM_005608	*PTPRCAP*	2.21	1.91	1.16
NM_003516.1	*HIST2H2AA3*	2.21	1.36	1.62
NM_001040874.1	*HIST2H2AA4*	2.21	1.36	1.62
NM_002167	*ID3*	2.20	4.96	-2.26
NM_182746	*MCM4*	2.20	2.39	-1.09
NM_000889	*ITGB7*	2.19	2.71	-1.24
NM_001258320	*TP53I11*	2.19	1.32	1.66
NM_178448	*SAPCD2*	2.19	2.69	-1.23
NR_033815	*ALS2CL*	2.17	4.98	-2.30
NM_003457	*ZNF207*	2.17	1.52	1.43
NR_045006	*NRON*	2.17	2.53	-1.17
NM_001258390	*LAYN*	2.16	3.42	-1.58

NM_201442	*C1S*	2.16	1.90	1.14
NM_001003697	*ATP5J*	2.16	-1.33	2.87
NM_001162497	*LPAR6*	2.16	3.16	-1.46
NM_080655	*MSANTD3*	2.16	1.82	1.18
NM_001145295	*NGLY1*	2.16	1.59	1.36
NM_001145319	*PLS1*	2.16	2.81	-1.31
NM_133279	*FCAR*	2.15	2.15	1.00
NM_016321	*RHCG*	2.15	7.23	-3.36
NR_047648	*FCGR2C*	2.15	-1.36	2.91
NR_038885	*LOC100506385*	2.15	1.78	1.20
NM_003979	*GPRC5A*	2.15	2.00	1.07
NM_014326	*DAPK2*	2.15	3.30	-1.54
NM_176871	*PDLIM2*	2.14	1.51	1.42
NM_007074	*CORO1A*	2.14	1.92	1.12
NM_001761	*CCNF*	2.14	2.57	-1.20
NM_017858	*TIPIN*	2.14	1.38	1.54
NM_001144897	*CD209*	2.14	4.64	-2.17
NM_001252103	*KIF21B*	2.13	2.73	-1.28
NR_037608	*SLX1A-*	2.13	-1.11	2.36
NR_037609	*SLX1B-*	2.13	-1.11	2.36
NR_033801	*SLC9A7P1*	2.12	5.69	-2.68
NM_002357	*MXD1*	2.12	2.43	-1.14
NM_033625	*RPL34*	2.12	-1.24	2.63
NM_001136031	*ATG7*	2.11	1.97	1.07
NR_027467	*LIMS3*	2.11	1.95	1.08
NR_038099	*LIMS3L*	2.11	1.95	1.08
NM_002916	*RFC4*	2.11	1.54	1.37
NM_001274	*CHEK1*	2.11	2.19	-1.04
NM_032867	*MICALCL*	2.11	1.25	1.69
NM_001009936	*PHF19*	2.10	2.10	1.00
NM_024807	*TREML2*	2.09	3.46	-1.65
NM_001177996	*FAM109A*	2.09	3.24	-1.55
NM_018455	*CENPN*	2.08	2.06	1.01
NM_024061	*ZNF655*	2.08	1.31	1.59
NM_001164489	*ADAM8*	2.08	1.06	1.97
NM_001190482	*PCSK5*	2.08	5.20	-2.50
NM_002183.1	*IL3RA*	2.08	3.89	-1.87
NM_152329	*LRR1*	2.08	1.05	1.99
NM_001177676	*GPR68*	2.07	3.94	-1.90
NM_203418	*RCAN1*	2.06	1.59	1.30

NM_173564	*NYAP1*	2.06	2.06	1.00
NM_006200	*PCSK5*	2.06	4.26	-2.07
NM_001682	*ATP2B1*	2.06	2.89	-1.40
NM_005539	*INPP5A*	2.06	1.51	1.36
NM_004834	*MAP4K4*	2.05	2.57	-1.25
NM_001083908	*DNAAF2*	2.05	-1.45	2.97
NM_138983	*OLIG1*	2.05	3.15	-1.54
NM_000064	*C3*	2.05	4.20	-2.05
NM_001012634	*IL32*	2.04	1.60	1.27
NM_014971	*EFR3B*	2.04	5.38	-2.63
NM_000376	*VDR*	2.04	2.86	-1.40
NM_001164751	*ASPH*	2.04	-1.32	2.70
NM_001193621	*PINLYP*	2.04	3.12	-1.54
NM_173341	*PHF7*	2.03	1.12	1.82
NM_001199382	*RNF145*	2.03	-1.48	3.01
NM_014448	*ARHGEF16*	2.03	2.15	-1.06
NM_004415	*DSP*	2.03	6.09	-3.00
NM_004186	*SEMA3F*	2.03	2.03	1.00
NM_002528	*NTHL1*	2.03	2.45	-1.21
NM_001185074	*ZCCHC6*	2.02	2.53	-1.25
NM_001012759	*CTU2*	2.02	1.34	1.51
NR_073090	*MEAF6*	2.02	2.60	-1.29
NM_152286	*PNPLA7*	2.02	3.34	-1.66
NM_005875	*EIF1B*	2.01	1.85	1.09
NM_003786	*ABCC3*	2.01	2.80	-1.39
NM_003897	*IER3*	2.01	1.51	1.34
NM_005491	*MAMLD1*	2.01	6.42	-3.19
NM_001142761	*KNSTRN*	2.01	2.21	-1.10
NM_015723	*PNPLA8*	2.01	2.90	-1.45
NM_001258210	*TSKU*	2.00	7.12	-3.55
NM_000903	*NQO1*	2.00	1.47	1.36
NM_001128826	*NCS1*	2.00	2.69	-1.34
NM_001126111	*OSGIN2*	2.00	2.16	-1.08
NM_023948	*MOSPD3*	1.99	2.59	-1.30
NM_003693	*SCARF1*	1.99	2.62	-1.32
NM_002183	*IL3RA*	1.99	3.86	-1.94
NM_001032283	*TMPO*	1.98	2.69	-1.36
NM_001029864	*KIAA1755*	1.97	6.95	-3.53
NM_001126104	*RACGAP1*	1.96	2.34	-1.19
NM_001943	*DSG2*	1.96	2.95	-1.51

NM_001242559	*MAP4K4*	1.94	3.27	-1.68
NM_153690	*FAM43A*	1.94	4.49	-2.31
NR_036440	*POU5F1P3*	1.94	2.11	-1.09
NM_001712	*CEACAM1*	1.94	4.04	-2.08
NM_001013838	*RLTPR*	1.94	2.85	-1.47
NM_000435	*NOTCH3*	1.93	5.99	-3.10
NM_174955	*ATP2A3*	1.93	3.19	-1.66
NM_006000	*TUBA4A*	1.92	2.70	-1.40
NM_016250	*NDRG2*	1.92	2.43	-1.27
NM_001005176	*SP140*	1.92	2.28	-1.19
NM_017596	*KIF21B*	1.91	2.59	-1.35
NM_001827	*CKS2*	1.90	2.47	-1.30
NM_001271856	*GRASP*	1.90	3.55	-1.86
NM_007028.7	*TRIM31*	1.90	7.84	-4.13
NM_001130026	*FAM115C*	1.90	-2.55	4.84
NM_014468	*VENTX*	1.90	2.23	-1.18
NM_020343	*RALGAPA2*	1.89	2.37	-1.25
NM_003276	*TMPO*	1.89	2.47	-1.31
NM_001099286	*MTFR2*	1.89	2.25	-1.19
NM_174954	*ATP2A3*	1.88	3.24	-1.72
NM_173528	*C15orf26*	1.88	2.17	-1.15
NM_001017535	*VDR*	1.87	2.80	-1.50
NM_199002	*ARHGEF1*	1.87	2.56	-1.37
NM_030929	*KAZALD1*	1.87	2.47	-1.32
NM_001135242	*NDRG1*	1.86	2.77	-1.49
NM_002438.1	*MRC1*	1.86	7.35	-3.96
NM_018387	*STRBP*	1.86	4.03	-2.17
NM_001252100	*KIF21B*	1.86	2.16	-1.17
NM_006716	*DBF4*	1.85	2.26	-1.22
NM_005173	*ATP2A3*	1.85	2.60	-1.41
NM_000379	*XDH*	1.84	3.07	-1.66
NM_004418	*DUSP2*	1.84	5.11	-2.77
NM_001256105	*WNT5A*	1.84	3.18	-1.73
NM_004844	*SH3BP5*	1.84	3.08	-1.68
NR_073104	*ITGA2*	1.84	2.34	-1.27
NM_001270691	*SMOX*	1.84	2.75	-1.50
NM_001037330	*TRIM16L*	1.83	2.21	-1.21
NM_174953	*ATP2A3*	1.83	3.14	-1.71
NM_001008723	*CCDC147*	1.82	2.04	-1.12
NM_033105	*DNAJC5B*	1.82	3.33	-1.83

NM_001145525	*RAI14*	1.82	2.47	-1.35
NM_002162	*ICAM3*	1.82	4.32	-2.37
NM_174956	*ATP2A3*	1.82	3.01	-1.65
NM_033141	*MAP3K9*	1.81	2.45	-1.35
NM_032744	*ADTRP*	1.81	2.68	-1.48
NM_001003702	*ARHGEF35*	1.81	3.68	-2.03
NM_025153	*ATP10B*	1.81	2.78	-1.54
NM_152292	*TRMT10A*	1.80	2.41	-1.34
NM_000906	*NPR1*	1.79	4.55	-2.54
NM_001079882	*PRKD2*	1.79	2.38	-1.33
NM_020698	*TMCC3*	1.79	3.56	-2.00
NM_015111	*N4BP3*	1.78	3.33	-1.87
NM_002933	*RNASE1*	1.78	-11.59	20.65
NM_001128922	*LRRC32*	1.78	6.97	-3.92
NM_004252	*SLC9A3R1*	1.78	2.12	-1.19
NM_003174	*SVIL*	1.78	3.03	-1.71
NM_002438	*MRC1*	1.78	7.77	-4.37
NM_181640	*CKLF*	1.76	2.01	-1.14
NM_001018009	*SH3BP5*	1.76	3.27	-1.86
NM_001161573	*MAFF*	1.76	2.25	-1.28
NM_001100164	*PHACTR2*	1.75	2.02	-1.15
NM_001628	*AKR1B1*	1.75	2.18	-1.24
NM_002981	*CCL1*	1.75	15.21	-8.71
NM_017705	*PAQR5*	1.75	2.72	-1.56
NM_001040457	*RHBDD2*	1.75	3.08	-1.76
NM_014059	*RGCC*	1.74	3.94	-2.26
NM_182705	*FAM101B*	1.74	4.31	-2.48
NR_073039	*MDK*	1.73	4.21	-2.43
NM_178452	*DNAAF1*	1.73	3.34	-1.93
NM_000920	*PC*	1.73	2.03	-1.17
NM_004878	*PTGES*	1.72	5.02	-2.92
NM_007237	*SP140*	1.71	2.29	-1.34
NM_002659	*PLAUR*	1.71	2.30	-1.35
NR_027237	*LOC728743*	1.71	2.50	-1.47
NM_001025195	*CES1*	1.71	7.20	-4.22
NM_006504	*PTPRE*	1.70	2.22	-1.31
NM_016246	*HSD17B14*	1.70	2.28	-1.34
NR_027383	*AKAP17A*	1.69	2.52	-1.49
NM_016951	*CKLF*	1.69	2.27	-1.34
NM_202001	*ERCC1*	1.68	2.20	-1.31

NM_145899	*HMGA1*	1.68	2.20	-1.31
NM_203434	*IER5L*	1.68	2.55	-1.52
NM_004669	*CLIC3*	1.68	2.25	-1.35
NM_018660	*ZNF395*	1.67	-2.00	3.36
NM_001076682	*NCAM1*	1.67	2.07	-1.24
NM_001024212	*S100A13*	1.67	3.97	-2.38
NM_002872	*RAC2*	1.66	4.24	-2.56
NM_015302	*HAUS5*	1.66	2.11	-1.27
NM_001945	*HBEGF*	1.65	6.67	-4.03
NM_032521	*PARD6B*	1.65	2.75	-1.67
NM_007317	*KIF22*	1.62	2.09	-1.29
NM_001130688	*HMGB2*	1.62	3.30	-2.04
NM_006866	*LILRA2*	1.62	2.62	-1.62
NM_001040716	*PC*	1.61	2.45	-1.52
NM_001135699	*YWHAZ*	1.61	2.11	-1.31
NM_001100819	*MOB4*	1.61	-2.25	3.62
NM_198275	*MPZL3*	1.61	2.70	-1.68
NM_000922	*PDE3B*	1.60	2.98	-1.86
NR_027157	*TMPO-AS1*	1.60	2.01	-1.25
NM_001134830	*AHI1*	1.60	3.87	-2.42
NM_001024809	*RARA*	1.60	2.33	-1.46
NM_001805	*CEBPE*	1.60	3.10	-1.94
NM_002990	*CCL22*	1.60	6.52	-4.08
NM_138434	*C7orf29*	1.59	3.11	-1.95
NM_053056	*CCND1*	1.59	2.56	-1.61
NM_006022	*TSC22D1*	1.59	2.19	-1.38
NM_000606	*C8G*	1.59	2.47	-1.55
NM_080725	*SRXN1*	1.59	2.01	-1.27
NM_012216	*MID2*	1.58	4.46	-2.82
NM_014210	*EVI2A*	1.58	2.46	-1.56
NM_001136050	*DHRS1*	1.58	2.16	-1.37
NM_018690	*APOBR*	1.57	2.83	-1.80
NM_001025159	*CD74*	1.57	2.20	-1.41
NM_022481	*ARAP3*	1.56	3.05	-1.95
NM_006599	*NFAT5*	1.56	2.36	-1.51
NM_152445	*FAM161B*	1.56	2.22	-1.42
NM_001145426	*CSDA*	1.56	-2.67	4.17
NM_024761	*MOB3B*	1.56	2.20	-1.41
NM_004900	*APOBEC3B*	1.56	3.32	-2.13
NM_178229	*IQGAP3*	1.55	2.41	-1.55

NM_006404	*PROCR*	1.55	2.18	-1.41
NM_002738	*PRKCB*	1.55	2.35	-1.52
NM_172037	*RDH10*	1.55	2.37	-1.53
NM_000804	*FOLR3*	1.55	2.62	-1.69
NM_003364	*UPP1*	1.55	2.21	-1.43
NM_001823	*CKB*	1.54	2.79	-1.80
NM_004951	*GPR183*	1.54	-2.12	3.27
NM_197947	*CLEC7A*	1.53	2.30	-1.51
NM_000103	*CYP19A1*	1.53	2.68	-1.76
NM_005417	*SRC*	1.53	2.00	-1.31
NM_002569	*FURIN*	1.52	2.03	-1.33
NM_002192	*INHBA*	1.50	3.61	-2.40
NM_138639	*BCL2L12*	1.50	2.24	-1.49
NM_003749	*IRS2*	1.50	2.54	-1.69
NM_005498	*AP1M2*	1.50	5.10	-3.40
NM_000507	*FBP1*	1.50	5.89	-3.94
NM_003689	*AKR7A2*	1.49	2.18	-1.46
NM_018944	*MIS18A*	1.49	2.50	-1.67
NM_004355	*CD74*	1.49	2.08	-1.39
NM_001817	*CEACAM4*	1.49	-2.31	3.43
NM_019111.2	*HLA-DRA*	1.49	2.33	-1.57
NM_145109	*MAP2K3*	1.48	2.54	-1.71
NM_001947	*DUSP7*	1.48	2.71	-1.83
NM_004882	*CIR1*	1.48	2.13	-1.44
NM_004227	*CYTH3*	1.48	2.41	-1.63
NM_006403	*NEDD9*	1.48	3.51	-2.38
NM_024832	*RIN3*	1.47	2.02	-1.38
NR_038386	*LOC728537*	1.47	2.76	-1.88
NM_002754	*MAPK13*	1.47	2.10	-1.43
NM_001025194	*CES1*	1.46	5.81	-3.97
NM_003390	*WEE1*	1.46	2.70	-1.84
NM_002615	*SERPINF1*	1.46	-2.14	3.13
NR_028492	*CFL1P1*	1.46	2.85	-1.95
NM_003887	*ASAP2*	1.46	3.67	-2.51
NM_015516	*TSKU*	1.45	6.13	-4.22
NM_001206798	*PKM*	1.45	2.16	-1.49
NR_003276	*CES1P1*	1.44	3.15	-2.18
NM_024103	*SLC25A23*	1.44	2.66	-1.85
NM_001130982	*DYSF*	1.44	2.16	-1.50
NM_001145290	*SLC37A2*	1.43	4.68	-3.27

NM_017873	*ASB6*	1.43	2.01	-1.40
NM_053064	*GNG2*	1.43	5.02	-3.51
NM_022833	*FAM129B*	1.43	2.18	-1.53
NM_002125	*HLA-DRB5*	1.42	2.40	-1.69
NM_015864	*FAM65B*	1.41	3.14	-2.22
NM_020225	*STOX2*	1.41	2.41	-1.71
NM_007297	*BRCA1*	1.41	2.72	-1.93
NM_080548	*PTPN6*	1.40	2.49	-1.78
NM_001256153	*ALOX5*	1.40	5.47	-3.90
NM_002124.1	*HLA-DRB1*	1.40	2.21	-1.58
NM_022047	*DEF6*	1.40	2.48	-1.77
NM_152362	*TNFAIP8L1*	1.40	2.25	-1.60
NM_001198525	*TCF7L2*	1.40	4.73	-3.38
NM_017957	*EPN3*	1.40	2.06	-1.48
NM_014862	*ARNT2*	1.40	-4.11	5.74
NM_015104	*ATG2A*	1.39	2.19	-1.57
NM_001111307	*PDE4A*	1.39	3.88	-2.79
NM_001040118	*ARAP1*	1.39	2.17	-1.56
NM_001145033	*C11orf96*	1.39	-3.97	5.50
NM_000164	*GIPR*	1.38	4.79	-3.46
NM_032348	*MXRA8*	1.38	2.08	-1.51
NR_027136	*C1orf126*	1.38	2.91	-2.11
NM_212535	*PRKCB*	1.38	2.20	-1.59
NM_001167942	*TNFAIP8L1*	1.38	2.24	-1.63
NM_001127628	*FBP1*	1.38	7.40	-5.38
NM_000399	*EGR2*	1.38	2.70	-1.97
NM_016112	*PKD2L1*	1.38	2.88	-2.10
NM_020645	*NRIP3*	1.37	2.02	-1.47
NM_032047	*B3GNT5*	1.37	2.10	-1.53
NM_021965	*PGM5*	1.36	3.40	-2.49
NR_027673	*ADARB1*	1.36	-2.56	3.49
NM_004395	*DBN1*	1.36	3.43	-2.52
NM_001164540	*DISC1*	1.36	-2.06	2.80
NM_005231	*CTTN*	1.36	3.06	-2.26
NM_173214	*NFAT5*	1.35	2.07	-1.53
NM_002121.4	*HLA-DPB1*	1.35	2.39	-1.77
NM_018249	*CDK5RAP2*	1.35	2.13	-1.58
NM_001171	*ABCC6*	1.35	2.37	-1.76
NM_001248004	*ARNTL2*	1.35	-2.77	3.73
NM_000218	*KCNQ1*	1.35	2.85	-2.12

NM_001085465	*CTNND1*	1.35	2.14	-1.59
NM_080430	*SELM*	1.35	-2.29	3.08
NM_033554.1	*HLA-DPA1*	1.34	2.15	-1.60
NM_007057	*ZWINT*	1.34	3.30	-2.46
NM_001677	*ATP1B1*	1.33	2.50	-1.88
NM_001256849	*POLD1*	1.33	2.39	-1.80
NM_003596	*TPST1*	1.33	-2.89	3.84
NM_000433	*NCF2*	1.33	2.31	-1.74
NM_003036	*SKI*	1.32	2.36	-1.78
NR_046386	*UHRF2*	1.32	-2.23	2.95
NM_007030	*TPPP*	1.32	3.00	-2.27
NM_015376	*RASGRP3*	1.32	4.11	-3.11
NM_000067	*CA2*	1.32	4.90	-3.72
NM_001127398	*ERLEC1*	1.32	2.77	-2.10
NM_002084	*GPX3*	1.31	2.71	-2.06
NM_021724	*NR1D1*	1.31	2.51	-1.91
NM_001166276	*ARHGAP25*	1.31	3.62	-2.76
NM_032932	*RAB11FIP4*	1.31	2.71	-2.06
NM_005944	*CD200*	1.31	-3.17	4.16
NM_001270615	*DEDD2*	1.31	2.07	-1.58
NM_144635	*FAM131A*	1.31	2.36	-1.81
NM_003713	*PPAP2B*	1.31	2.08	-1.59
NM_024659	*GTDC1*	1.30	-2.45	3.20
NM_017421	*COQ3*	1.30	2.17	-1.67
NM_001267709	*ZNF706*	1.30	2.49	-1.91
NR_038111	*SNHG16*	1.30	2.16	-1.66
NM_001004105	*GRK6*	1.30	2.14	-1.65
NM_030776	*ZBP1*	1.29	4.68	-3.61
NM_001035254	*FAM102A*	1.29	2.28	-1.76
NM_001033555	*SPECC1*	1.29	2.85	-2.21
NR_027381	*RDH13*	1.28	2.65	-2.06
NM_175900	*C16orf54*	1.28	2.53	-1.97
NM_006495	*EVI2B*	1.28	2.08	-1.62
NM_002998	*SDC2*	1.28	2.34	-1.83
NM_001190789	*NCF2*	1.27	2.38	-1.87
NM_001220769	*IKZF1*	1.27	-2.00	2.54
NM_015627	*LDLRAP1*	1.27	2.05	-1.62
NM_145655	*GCNT2*	1.26	2.15	-1.70
NM_001271629	*MEF2D*	1.26	2.13	-1.68
NM_021070	*LTBP3*	1.26	3.59	-2.84

NM_002118.5	*HLA-DMB*	1.26	2.80	-2.23
NM_198044	*ZDHHC16*	1.26	-2.49	3.13
NM_001142928	*LRRC61*	1.25	2.08	-1.67
NM_001040217	*FAM63A*	1.25	2.12	-1.70
NM_001193484	*LIMS1*	1.25	2.02	-1.62
NM_001100624	*CENPN*	1.25	2.82	-2.27
NM_021224	*ZNF462*	1.25	-3.06	3.82
NM_016183	*MRTO4*	1.24	2.15	-1.73
NM_001190267	*ATG16L1*	1.24	-2.03	2.52
NM_020786	*PDP2*	1.24	2.43	-1.96
NM_001204108	*BCL2L11*	1.24	-2.76	3.42
NM_006456	*ST6GALNAC2*	1.24	2.64	-2.13
NM_177551	*HCAR2*	1.24	4.04	-3.27
NM_001242525.3	*HLA-DPA1*	1.24	2.14	-1.73
NR_015421	*LOC154761*	1.23	-4.14	5.09
NM_145263	*SPATA18*	1.23	2.77	-2.26
NM_133463	*AMZ1*	1.23	2.46	-2.01
NM_001266	*CES1*	1.22	5.35	-4.38
NM_002115	*HK3*	1.22	2.36	-1.93
NM_003955	*SOCS3*	1.22	-2.21	2.69
NM_001085464	*CTNND1*	1.22	2.21	-1.81
NM_001085463	*CTNND1*	1.21	2.30	-1.90
NR_051978	*TBC1D2*	1.21	2.38	-1.97
NM_001109974	*SYNPO*	1.21	-4.95	5.99
NR_052852	*MARCKSL1*	1.21	-2.45	2.96
NM_001206886	*CTNND1*	1.21	2.22	-1.84
NM_018843	*SLC25A40*	1.21	2.17	-1.80
NM_182898	*CREB5*	1.20	2.19	-1.82
NM_198595	*AFAP1*	1.20	-2.29	2.75
NM_001171088	*CLCN2*	1.20	2.84	-2.37
NR_047545	*LMNA*	1.19	2.33	-1.95
NM_001130420	*SMARCC2*	1.19	-2.16	2.58
NM_000638	*VTN*	1.19	-2.48	2.95
NM_033334	*NR6A1*	1.19	-2.89	3.44
NM_001206883	*CTNND1*	1.19	2.36	-1.99
NM_205842	*NCKAP1*	1.18	3.13	-2.64
NM_001270941	*JAKMIP2*	1.18	4.29	-3.64
NM_133261	*GIPC3*	1.17	2.77	-2.36
NM_001193523	*FAM65A*	1.17	2.10	-1.80
NM_181597	*UPP1*	1.17	2.11	-1.81

NM_022304	*HRH2*	1.16	-2.95	3.44
NM_006869	*ADAP1*	1.16	2.29	-1.98
NM_003214	*TEAD3*	1.16	4.53	-3.91
NM_213636	*PDLIM7*	1.16	2.71	-2.34
NM_001012968	*SPIN4*	1.16	2.85	-2.46
NR_003187	*NCF1C*	1.16	2.10	-1.82
NM_020443	*NAV1*	1.15	-2.53	2.92
NR_046336	*FAM193A*	1.15	-2.86	3.29
NM_012201	*GLG1*	1.14	-2.89	3.31
NM_005110	*GFPT2*	1.14	-6.72	7.66
NM_001878	*CRABP2*	1.14	7.56	-6.64
NM_004417	*DUSP1*	1.13	-2.10	2.38
NM_033254	*BOC*	1.13	-2.61	2.95
NM_203352	*PDLIM7*	1.13	2.51	-2.22
NM_001166271	*SPATA13*	1.13	4.49	-3.97
NM_001127651	*NCF2*	1.13	2.67	-2.36
NM_001145642	*KIAA0226*	1.13	2.28	-2.02
NM_002119.6	*HLA-DOA*	1.12	3.01	-2.69
NM_000239	*LYZ*	1.12	2.49	-2.23
NM_000265	*NCF1*	1.12	2.18	-1.96
NM_021922	*FANCE*	1.12	3.23	-2.89
NM_001033053	*NLRP1*	1.11	2.45	-2.20
NR_037669	*GGCT*	1.11	-2.43	2.69
NM_001256370	*SAMSN1*	1.11	-4.24	4.71
NM_014790	*JAKMIP2*	1.11	2.85	-2.57
NM_020219	*CEACAM19*	1.11	-2.65	2.94
NR_027118	*INHBA-AS1*	1.10	2.46	-2.23
NM_002914	*RFC2*	1.10	-2.50	2.73
NM_015316	*PPP1R13B*	1.09	2.50	-2.29
NM_171825	*CAMK2A*	1.08	-3.69	4.00
NM_003612	*SEMA7A*	1.08	4.41	-4.07
NM_001190794	*NCF2*	1.08	2.17	-2.01
NM_032855	*HSH2D*	1.08	2.11	-1.96
NM_006287	*TFPI*	1.07	-2.37	2.54
NM_001253837	*PKD2L1*	1.07	2.05	-1.91
NM_001080855	*PXN*	1.07	2.33	-2.18
NR_033842	*TMEM72-AS1*	1.07	-2.20	2.35
NM_000698	*ALOX5*	1.07	5.91	-5.54
NM_005451	*PDLIM7*	1.06	2.65	-2.49
NM_001270467	*SOCS2*	1.06	-3.75	3.97

NM_001171816	*RNF166*	1.06	2.07	-1.96
NM_032784	*RSPO3*	1.05	3.14	-2.98
NM_001197293	*DPYSL2*	1.05	-2.46	2.59
NM_001629	*ALOX5AP*	1.05	9.70	-9.26
NM_001135769	*PVR*	1.05	-2.54	2.66
NM_012413	*QPCT*	1.04	3.69	-3.53
NM_016518	*PIPOX*	1.04	3.56	-3.42
NM_001060	*TBXA2R*	1.03	2.48	-2.40
NM_001261414	*SOX5*	1.03	-3.42	3.53
NM_199070	*NDUFAF3*	1.03	-2.06	2.12
NM_138360	*LRRC16B*	1.03	3.88	-3.77
NM_001127244	*LRRC8A*	1.03	2.38	-2.31
NM_001725	*BPI*	1.03	2.91	-2.83
NM_001010855	*PIK3R6*	1.02	2.21	-2.15
NM_007021	*C10orf10*	1.02	-2.27	2.33
NM_001172699	*PPARGC1B*	1.02	2.62	-2.56
NM_005114	*HS3ST1*	1.02	-4.88	4.99
NM_006095	*ATP8A1*	1.02	-2.10	2.15
NM_173505	*ANKRD29*	1.01	2.13	-2.10
NM_001257281	*DIS3L2*	1.01	-2.33	2.36
NM_002610	*PDK1*	1.01	-2.24	2.26
NM_001135770	*PVR*	1.01	-2.35	2.37
NM_022136	*SAMSN1*	1.01	-2.67	2.70
NM_001267595	*SENP1*	1.01	-2.09	2.12
NM_181866	*ACOT7*	1.00	3.17	-3.16
NM_003890	*FCGBP*	1.00	-5.73	5.73
NM_182932	*SLC8A3*	1.00	-5.46	5.46
NM_005562	*LAMC2*	1.00	-4.69	4.69
NM_003064	*SLPI*	1.00	-4.09	4.09
NM_012153	*EHF*	1.00	-3.63	3.63
NM_004198	*CHRNA6*	1.00	-3.61	3.61
NM_003116	*SPAG4*	1.00	-2.88	2.88
NR_026656	*LOC400043*	1.00	-2.70	2.70
NM_052934	*SLC26A9*	1.00	-2.66	2.66
NM_001142480	*NREP*	1.00	-2.02	2.02
NM_001254952.1	*ATAT1*	-1.00	-2.41	2.41
NM_007144	*PCGF2*	-1.00	2.33	-2.34
NM_003118	*SPARC*	-1.01	2.34	-2.36
NM_015831	*ACHE*	-1.01	-6.00	5.94
NM_001177306	*PAM*	-1.01	-3.42	3.38

NM_004566	*PFKFB3*	-1.02	-4.39	4.32
NM_001098526	*AMICA1*	-1.02	2.27	-2.31
NM_006399	*BATF*	-1.02	-2.71	2.65
NM_013227	*ACAN*	-1.03	-2.72	2.65
NM_006343	*MERTK*	-1.03	-2.08	2.02
NM_001257291	*SLC9A7*	-1.03	2.48	-2.55
NM_181351	*NCAM1*	-1.03	3.25	-3.35
NM_003629	*PIK3R3*	-1.03	2.04	-2.10
NM_015981	*CAMK2A*	-1.03	-2.13	2.06
NM_012118	*CCRN4L*	-1.04	-2.18	2.10
NM_001199037	*SERINC2*	-1.04	2.23	-2.32
NM_001025239	*TSPAN4*	-1.05	-2.14	2.05
NM_004272	*HOMER1*	-1.05	2.03	-2.13
NM_138495	*ATXN7L1*	-1.05	-3.88	3.68
NM_006862	*TDRKH*	-1.05	-2.68	2.55
NM_000930	*PLAT*	-1.05	-5.44	5.16
NM_002185	*IL7R*	-1.06	-3.35	3.17
NM_001127596	*FCGR3A*	-1.06	-2.32	2.20
NM_138463	*TLCD1*	-1.06	-2.04	1.93
NM_005165	*ALDOC*	-1.06	-2.67	2.52
NM_018646	*TRPV6*	-1.06	-2.04	1.92
NM_198471	*KANK3*	-1.06	2.12	-2.25
NM_033006	*NLRP1*	-1.06	2.46	-2.62
NR_002186	*DKFZP586I142*	-1.07	-2.34	2.18
NM_001174066	*FGFR1*	-1.07	2.25	-2.41
NM_002828	*PTPN2*	-1.07	-2.44	2.28
NM_001025252	*TPD52*	-1.08	-4.00	3.69
NM_003651	*CSDA*	-1.08	-2.04	1.88
NM_173797	*PAPD4*	-1.08	-5.01	4.62
NM_014858	*TMCC2*	-1.09	-10.03	9.22
NM_032154	*PCGF6*	-1.09	2.06	-2.25
NM_004139	*LBP*	-1.10	-9.25	8.37
NM_002210	*ITGAV*	-1.11	-2.47	2.22
NM_002543	*OLR1*	-1.12	6.29	-7.01
NM_001270473	*CENPN*	-1.12	2.36	-2.63
NM_182493	*MYLK3*	-1.12	-3.25	2.90
NR_027716	*H2AFJ*	-1.12	-2.63	2.34
NR_024621	*C11orf21*	-1.13	2.44	-2.75
NM_002318	*LOXL2*	-1.13	-2.78	2.47
NM_000891	*KCNJ2*	-1.13	-2.63	2.33

NM_001267822	*ZMYND15*	-1.13	2.28	-2.57
NM_001145000	*ITGAV*	-1.13	-2.99	2.64
NM_152342	*CDYL2*	-1.13	-2.55	2.25
NM_014344	*FJX1*	-1.13	-2.10	1.85
NM_001256579	*LOC388813*	-1.13	-4.31	3.80
NR_040079	*LOC399715*	-1.13	-2.82	2.49
NM_001459	*FLT3LG*	-1.13	-3.09	2.72
NM_206824	*VKORC1*	-1.14	-3.46	3.04
NM_004052	*BNIP3*	-1.14	-4.55	4.00
NM_001244701	*ZFP36L1*	-1.14	-2.57	2.26
NM_001145305	*KIAA1683*	-1.14	-2.29	2.01
NM_002885	*RAP1GAP*	-1.14	2.86	-3.28
NM_002196	*INSM1*	-1.15	-2.83	2.46
NM_001254738	*RND3*	-1.15	-4.33	3.76
NM_015259	*ICOSLG*	-1.16	3.19	-3.69
NM_001131055	*HRH2*	-1.16	-8.01	6.88
NR_027011	*CSDAP1*	-1.16	-2.17	1.86
NM_001242877	*ELP2*	-1.17	-3.34	2.87
NM_001271007	*STRA13*	-1.17	-2.70	2.32
NM_001080976	*DSE*	-1.17	-2.18	1.86
NM_001256154	*ALOX5*	-1.17	4.05	-4.74
NM_133631	*ROBO1*	-1.17	-6.32	5.40
NM_001173488	*NKRF*	-1.17	-2.62	2.24
NM_001040655	*TTC23*	-1.17	2.88	-3.37
NM_020717	*SHROOM4*	-1.17	2.45	-2.88
NM_001145903.1	*C2*	-1.17	-2.15	1.83
NM_153259	*MCOLN2*	-1.18	-2.03	1.73
NR_026589	*WHAMMP2*	-1.18	-2.34	1.99
NM_001710	*CFB*	-1.18	-2.09	1.78
NM_020704	*STRIP2*	-1.18	-3.25	2.75
NM_001165	*BIRC3*	-1.18	-2.69	2.27
NM_001167985	*INCA1*	-1.18	-2.42	2.05
NR_026859	*ULK4P3*	-1.19	-2.28	1.92
NM_012334	*MYO10*	-1.19	-3.27	2.75
NM_175052	*ST8SIA4*	-1.19	-2.29	1.93
NM_001199887	*IL7*	-1.19	-2.72	2.28
NM_003775	*S1PR4*	-1.19	2.22	-2.64
NM_003812	*ADAM23*	-1.19	-3.48	2.92
NM_001759	*CCND2*	-1.20	3.87	-4.63
NM_000651	*CR1*	-1.20	-5.82	4.85

NM_001164234	*DDHD2*	-1.20	-2.08	1.73
NR_072980	*NIM1*	-1.20	-2.18	1.81
NM_003485	*GPR68*	-1.21	3.16	-3.81
NM_001001547	*CD36*	-1.21	-3.18	2.63
NM_001040668	*BCL2L12*	-1.21	-3.08	2.54
NM_001170631	*FCAMR*	-1.21	-15.66	12.91
NM_002356	*MARCKS*	-1.22	-2.34	1.91
NM_145172	*WDR63*	-1.22	-2.38	1.95
NM_001161357	*FCHO1*	-1.23	2.08	-2.55
NM_001172509	*SATB2*	-1.23	-2.52	2.05
NM_033211	*C5orf30*	-1.23	-2.07	1.69
NM_005512	*LRRC32*	-1.23	2.65	-3.26
NR_038902	*JAKMIP2-AS1*	-1.23	2.56	-3.15
NR_028050	*MTERFD2*	-1.23	-3.20	2.60
NM_181726	*ANKRD37*	-1.24	-3.48	2.82
NM_001164469	*TMED7-*	-1.24	-2.20	1.77
NM_014945	*ABLIM3*	-1.25	-3.27	2.62
NM_014883	*FAM13A*	-1.25	-2.21	1.77
NM_001243778	*FAM213A*	-1.25	2.28	-2.85
NM_080625	*CCM2L*	-1.25	-3.84	3.08
NM_147187	*TNFRSF10B*	-1.25	-2.07	1.65
NM_001171159	*SIGLEC10*	-1.25	-2.19	1.74
NM_206954	*PRAME*	-1.25	-3.37	2.69
NM_181655	*C17orf58*	-1.26	-2.87	2.28
NM_057180	*VPS29*	-1.26	-2.33	1.85
NM_001024946	*ASL*	-1.27	-5.25	4.15
NM_003560	*PLA2G6*	-1.28	-2.16	1.69
NM_001198689	*LEPR*	-1.28	-2.11	1.65
NM_000665	*ACHE*	-1.28	-12.39	9.66
NM_021958	*HLX*	-1.28	-2.44	1.90
NM_006092	*NOD1*	-1.29	-2.36	1.84
NM_130384	*ATRIP*	-1.29	-2.24	1.74
NM_018565	*SERINC2*	-1.29	4.22	-5.45
NM_007173	*PRSS23*	-1.29	-2.82	2.19
NM_006955	*ZNF33B*	-1.29	-2.04	1.58
NM_001165414	*LDHA*	-1.29	-2.46	1.90
NM_001142483	*NREP*	-1.29	-2.28	1.76
NM_006505	*PVR*	-1.29	-2.76	2.13
NM_000875	*IGF1R*	-1.30	-2.05	1.59
NM_014824	*FCHSD2*	-1.30	-2.18	1.67

NM_013276	*SHPK*	-1.30	2.21	-2.88
NM_153634	*CPNE8*	-1.30	-2.04	1.57
NM_001100117	*RIMS2*	-1.30	-2.98	2.29
NM_139022	*TSPAN32*	-1.30	2.19	-2.85
NM_001145658	*RAP1GAP*	-1.30	3.21	-4.18
NM_000020	*ACVRL1*	-1.31	2.20	-2.89
NM_153746	*ZDHHC14*	-1.31	-2.06	1.57
NM_014791	*MELK*	-1.31	-2.76	2.10
NM_001035005	*C18orf32*	-1.32	-2.15	1.63
NM_001184717	*TIPARP*	-1.33	-3.03	2.28
NM_030668	*PTPRO*	-1.33	-2.07	1.55
NM_032148	*SLC41A2*	-1.33	-2.12	1.59
NM_007222	*ZHX1*	-1.34	-2.34	1.75
NM_002424	*MMP8*	-1.34	-3.50	2.61
NR_040086	*SETD4*	-1.34	2.16	-2.90
NM_001206609	*SELPLG*	-1.35	-2.25	1.67
NR_026806	*FLJ13224*	-1.35	-2.03	1.51
NM_000088	*COL1A1*	-1.35	-3.98	2.95
NM_147128	*ZNRF2*	-1.35	-3.06	2.27
NM_001207013	*PTPN2*	-1.35	-3.80	2.81
NM_203288	*RP9*	-1.35	-2.20	1.63
NM_004099	*STOM*	-1.35	-2.24	1.65
NM_207360	*ZC3H12D*	-1.36	-2.12	1.57
NM_001466	*FZD2*	-1.36	-3.26	2.40
NR_047701	*COA1*	-1.36	-2.41	1.77
NM_144683	*DHRS13*	-1.36	-3.20	2.35
NM_005668	*ST8SIA4*	-1.36	-2.66	1.95
NR_038863	*LOC285758*	-1.37	-4.48	3.28
NM_005715	*UST*	-1.37	-2.19	1.60
NR_003334	*SNORD116-20*	-1.37	-2.01	1.47
NR_002980	*SNORA50*	-1.38	-2.28	1.66
NM_005090	*JMJD7-*	-1.38	-2.38	1.73
NM_003006	*SELPLG*	-1.38	-2.19	1.58
NM_194278	*ELMSAN1*	-1.38	-2.03	1.47
NM_000712	*BLVRA*	-1.38	-2.30	1.66
NM_138636	*TLR8*	-1.39	-2.06	1.48
NM_021991	*JUP*	-1.40	2.14	-2.99
NM_001112800	*SLC8A1*	-1.40	-2.17	1.55
NR_040101	*KLHDC3*	-1.41	-2.25	1.60
NM_001134707	*SARDH*	-1.41	3.63	-5.10

NM_025263.1	*PRR3*	-1.41	-2.48	1.76
NM_021097	*SLC8A1*	-1.41	-2.16	1.54
NM_153235	*TXLNB*	-1.41	-2.00	1.42
NM_199436	*SPAST*	-1.41	-2.48	1.75
NM_032023	*RASSF4*	-1.41	-3.61	2.55
NM_016108	*AIG1*	-1.41	-2.74	1.94
NM_001128210	*SPRED2*	-1.42	-3.35	2.36
NM_032143	*ZRANB3*	-1.42	-2.16	1.52
NM_001261829	*MINA*	-1.43	2.48	-3.55
NR_002983	*SNORA55*	-1.44	-2.43	1.69
NR_024110	*SBDSP1*	-1.44	-2.42	1.68
NM_203463	*CERS6*	-1.44	-2.83	1.96
NR_000005	*SNORD15A*	-1.45	-2.26	1.56
NM_006206	*PDGFRA*	-1.45	-2.37	1.63
NM_012329	*MMD*	-1.45	-2.30	1.58
NM_001207019	*FCER2*	-1.46	2.90	-4.23
NM_007261	*CD300A*	-1.46	-2.67	1.82
NR_038123	*PSMA3*	-1.47	-2.75	1.87
NM_005099	*ADAMTS4*	-1.47	-35.27	23.95
NM_015869	*PPARG*	-1.48	2.22	-3.29
NM_005688	*ABCC5*	-1.48	-2.11	1.42
NM_020845	*PITPNM2*	-1.48	-2.79	1.88
NM_004332	*BPHL*	-1.49	-2.52	1.70
NM_002060	*GJA4*	-1.49	-5.54	3.73
NM_001127700	*SERPINA1*	-1.49	-2.53	1.69
NM_001040659	*TTC23*	-1.50	-3.17	2.10
NR_003697	*SNHG15*	-1.52	-2.68	1.77
NM_001039660	*IL18BP*	-1.52	-2.21	1.46
NR_038304	*LOC100505702*	-1.52	-3.24	2.13
NM_001001391	*CD44*	-1.52	-2.12	1.39
NM_001242638	*IL31RA*	-1.52	-3.15	2.07
NM_025239	*PDCD1LG2*	-1.53	-2.34	1.53
NM_020179	*C11orf75*	-1.53	-2.05	1.34
NM_003632	*CNTNAP1*	-1.53	-2.58	1.69
NM_201436	*H2AFV*	-1.53	-3.99	2.60
NM_001114395	*CNTLN*	-1.54	-2.80	1.82
NM_001003679	*LEPR*	-1.55	-2.38	1.54
NR_003079	*SNORD111*	-1.57	-2.72	1.73
NM_001168241	*GAREML*	-1.57	-2.10	1.33
NM_001201362	*HMGN3*	-1.58	-2.04	1.30

NM_178562	*TSPAN33*	-1.58	-3.68	2.32
NM_000643	*AGL*	-1.58	-2.05	1.30
NM_003655	*CBX4*	-1.59	-2.70	1.70
NR_024405	*LOC730101*	-1.59	-2.61	1.65
NM_172002	*HSCB*	-1.59	-2.14	1.35
NM_080422	*PTPN2*	-1.59	-3.13	1.97
NM_002987	*CCL17*	-1.59	-5.50	3.45
NM_004192	*ASMTL*	-1.59	-2.62	1.65
NM_000789	*ACE*	-1.60	2.57	-4.10
NM_001167604	*XPNPEP1*	-1.61	-2.05	1.28
NM_001193657	*C17orf62*	-1.61	-2.56	1.59
NM_001723	*DST*	-1.61	-2.46	1.52
NM_001244897	*PTBP3*	-1.62	-2.13	1.31
NM_030958	*SLCO5A1*	-1.62	-2.94	1.82
NM_023009	*MARCKSL1*	-1.62	-3.78	2.33
NM_001010917	*GOLGA7B*	-1.62	-2.47	1.52
NM_001031711	*ERGIC1*	-1.62	-2.37	1.46
NM_007270	*FKBP9*	-1.62	-2.10	1.29
NM_001242370	*CACFD1*	-1.63	-2.31	1.42
NR_045690	*C19orf12*	-1.63	-2.51	1.55
NM_014333	*CADM1*	-1.63	-2.67	1.64
NM_001080824	*TRABD2A*	-1.63	2.90	-4.73
NM_003195	*TCEA2*	-1.63	-2.00	1.23
NM_001637	*AOAH*	-1.64	-2.92	1.79
NM_001018037	*VPS13A*	-1.64	-2.20	1.34
NM_152904	*SPECC1*	-1.64	-4.05	2.47
NM_052818	*N4BP2L1*	-1.65	-2.54	1.54
NM_006202	*PDE4A*	-1.65	2.06	-3.39
NM_015957	*APIP*	-1.65	-2.05	1.24
NM_000240	*MAOA*	-1.66	-16.58	10.01
NM_002390	*ADAM11*	-1.66	-3.76	2.27
NM_001144999	*ITGAV*	-1.66	-3.53	2.13
NM_001174152	*RABEPK*	-1.66	-4.46	2.68
NM_015675	*GADD45B*	-1.66	-2.00	1.21
NM_006829	*C10orf116*	-1.67	-3.28	1.97
NM_001386	*DPYSL2*	-1.67	-2.52	1.51
NM_001033553	*SPECC1*	-1.67	-2.38	1.42
NM_001037442	*RUFY3*	-1.67	-2.12	1.26
NM_004466	*GPC5*	-1.67	-2.93	1.75
NM_176894	*P2RY13*	-1.68	-7.60	4.52

NM_001145515	*SCRN1*	-1.68	-3.50	2.08
NM_004273	*CHST3*	-1.68	2.65	-4.45
NR_003331	*SNORD116-16*	-1.68	-3.28	1.94
NM_001009944	*PKD1*	-1.69	-2.14	1.27
NM_017923	*1-Mar*	-1.69	-4.53	2.68
NM_173653	*SLC9A9*	-1.69	-2.63	1.55
NM_001243247	*NPRL3*	-1.69	-2.24	1.32
NR_037918	*PRH1-PRR4*	-1.70	-2.11	1.24
NR_028371	*FAS-AS1*	-1.70	-2.48	1.46
NM_001242925	*TMCC2*	-1.70	-11.70	6.88
NM_001142573	*IMPDH1*	-1.70	-3.07	1.80
NM_001110514	*EBF4*	-1.70	-8.77	5.15
NM_173607	*FAM177A1*	-1.70	-2.09	1.23
NM_005655	*KLF10*	-1.71	-2.01	1.18
NM_001010919	*FAM26F*	-1.71	-3.71	2.17
NM_139047	*MAPK8*	-1.72	-2.64	1.54
NM_001173540	*BANP*	-1.72	2.02	-3.47
NM_173206	*PIAS2*	-1.72	-2.05	1.19
NM_003730	*RNASET2*	-1.73	-2.53	1.47
NM_001112801	*SLC8A1*	-1.73	-2.30	1.33
NM_001099625	*MTFR1L*	-1.73	-2.05	1.18
NM_002751	*MAPK11*	-1.73	-2.57	1.49
NR_003336	*SNORD116-22*	-1.73	-2.99	1.73
NR_003318	*SNORD116-3*	-1.73	-2.44	1.41
NR_003324	*SNORD116-9*	-1.73	-2.44	1.41
NM_001256126	*CERS6*	-1.74	-3.57	2.06
NM_001193646	*ATF5*	-1.75	-2.10	1.21
NM_001146289	*LEPRE1*	-1.75	-2.08	1.19
NM_001128913	*PCBP2*	-1.75	-2.47	1.41
NM_001924	*GADD45A*	-1.75	-5.32	3.04
NM_003877	*SOCS2*	-1.75	-5.65	3.22
NM_001243439	*SPECC1*	-1.76	-2.59	1.47
NM_001195227	*TRPM4*	-1.76	-2.16	1.23
NR_049764	*FBXO28*	-1.76	2.83	-4.98
NM_001135662	*RAB7L1*	-1.77	-2.50	1.41
NR_038843	*GOLGA8S*	-1.77	-2.38	1.34
NR_003521	*WHAMMP3*	-1.77	-2.78	1.57
NM_001252624	*SLC8A1*	-1.78	-2.30	1.30
NM_032898	*CEP19*	-1.78	-2.50	1.41
NM_001243351	*NUB1*	-1.78	-2.27	1.28

NR_033933	*GOLGA8T*	-1.79	-2.31	1.30
NM_001289	*CLIC2*	-1.79	-2.93	1.64
NM_001127443	*CD36*	-1.79	-6.34	3.55
NM_014395	*DAPP1*	-1.79	-3.09	1.72
NM_021903.4	*GABBR1*	-1.80	-2.18	1.21
NM_018224	*COA1*	-1.81	-3.16	1.75
NM_006985	*NPIP*	-1.81	-2.16	1.19
NM_018927	*PCDHGB7*	-1.81	-2.68	1.48
NM_001001548	*CD36*	-1.82	-4.75	2.62
NM_172160	*KCNAB1*	-1.82	-2.15	1.18
NM_144590	*ANKRD22*	-1.82	-2.67	1.47
NM_004710	*SYNGR2*	-1.83	-2.24	1.22
NM_002969	*MAPK12*	-1.83	-2.35	1.28
NM_001270471	*SOCS2*	-1.84	-9.48	5.16
NM_014961	*RUFY3*	-1.84	-2.41	1.31
NM_173042	*IL18BP*	-1.84	-2.62	1.42
NM_001171156	*SIGLEC10*	-1.84	-2.48	1.35
NM_001204450	*CCPG1*	-1.85	-2.07	1.12
NM_194328	*RNF38*	-1.85	-2.06	1.11
NM_001135239	*LDHA*	-1.85	-3.33	1.80
NM_001540	*HSPB1*	-1.85	-2.25	1.22
NM_016118	*NUB1*	-1.85	-2.30	1.24
NR_046478	*FKBP14*	-1.86	-4.20	2.26
NM_001135161	*COMT*	-1.87	-2.28	1.22
NM_004086	*COCH*	-1.89	-3.33	1.76
NM_138799	*MBOAT2*	-1.89	-3.39	1.79
NM_001261441	*EXTL2*	-1.90	-2.48	1.30
NR_003317	*SNORD116-2*	-1.91	-2.29	1.20
NM_021111	*RECK*	-1.91	-2.12	1.11
NM_021129	*PPA1*	-1.92	-5.81	3.02
NM_002349	*LY75*	-1.93	-2.37	1.23
NM_003644	*GAS7*	-1.94	-2.40	1.24
NM_001258207	*VAV1*	-1.94	-3.14	1.62
NM_001126118	*TP53*	-1.95	-5.08	2.60
NM_173664	*ARL10*	-1.96	-2.68	1.37
NM_002404	*MFAP4*	-1.96	-2.06	1.05
NM_198572	*SPATC1*	-1.97	-4.16	2.12
NM_004560	*ROR2*	-1.97	-2.75	1.39
NM_001177506	*AOAH*	-1.97	-3.76	1.91
NM_005962	*MXI1*	-1.98	-5.12	2.59

NM_020739	*CCPG1*	-1.98	-2.31	1.17
NM_017738	*CNTLN*	-1.98	-2.46	1.24
NM_001123383	*BCOR*	-1.98	-2.58	1.30
NM_005755	*EBI3*	-1.98	-3.18	1.60
NM_000958	*PTGER4*	-1.99	-2.31	1.16
NM_001024943	*ASL*	-1.99	-2.55	1.28
NM_000296	*PKD1*	-1.99	-2.74	1.38
NM_001042410	*ANKZF1*	-2.00	-4.17	2.09
NM_001207049	*BACE1*	-2.00	-2.34	1.17
NM_005170	*ASCL2*	-2.00	-1.52	-1.32
NM_152400	*C4orf32*	-2.00	-2.01	1.00
NM_001009933	*DNASE1L1*	-2.01	-1.99	-1.01
NR_003323	*SNORD116-8*	-2.01	-2.28	1.14
NM_001552	*IGFBP4*	-2.01	-1.23	-1.63
NM_020142	*NDUFA4L2*	-2.01	-2.10	1.05
NM_000962	*PTGS1*	-2.01	-1.22	-1.65
NM_001103167	*ZGLP1*	-2.01	-1.77	-1.14
NM_024490	*ATP10A*	-2.01	-2.19	1.09
NM_001256394	*NOL8*	-2.01	-2.02	1.00
NM_015548	*DST*	-2.01	-2.85	1.41
NM_001172412	*VANGL1*	-2.01	-1.87	-1.08
NM_003045	*SLC7A1*	-2.01	-1.94	-1.04
NM_001170931	*FOXO4*	-2.02	1.18	-2.38
NM_173657	*C3orf33*	-2.02	-1.23	-1.64
NM_001025235	*TSPAN4*	-2.02	-3.62	1.79
NM_001112802	*SLC8A1*	-2.02	-2.48	1.23
NM_152989	*SOX5*	-2.02	-5.37	2.66
NR_024117.1	*MSTO2P*	-2.02	-1.76	-1.15
NM_014666	*CLINT1*	-2.03	-1.46	-1.38
NR_003337	*SNORD116-23*	-2.03	-2.64	1.30
NM_007286	*SYNPO*	-2.03	-9.96	4.90
NM_015517	*HINFP*	-2.03	-2.22	1.09
NM_018649	*H2AFY2*	-2.04	-2.44	1.20
NM_004529	*MLLT3*	-2.05	-3.98	1.94
NM_201612	*IKBIP*	-2.05	-3.20	1.56
NM_024296	*CCDC28B*	-2.05	-2.17	1.06
NR_002936	*TOB2P1*	-2.06	-1.55	-1.32
NR_046329	*TRIM46*	-2.06	-1.66	-1.24
NM_001127444	*CD36*	-2.06	-5.81	2.82
NM_006039	*MRC2*	-2.06	-1.48	-1.39

NM_003741	*CHRD*	-2.06	1.53	-3.16
NM_001207018	*SMTN*	-2.06	-2.93	1.42
NM_006274	*CCL19*	-2.07	-37.30	18.02
NR_003340	*SNORD116-26*	-2.07	-2.31	1.12
NR_047546	*SCFD1*	-2.07	-1.10	-1.88
NM_032276	*RHBDD1*	-2.07	-1.87	-1.11
NM_017549	*EPDR1*	-2.07	-2.95	1.42
NM_001130709	*RUFY3*	-2.07	-3.57	1.72
NM_001144936	*C11orf95*	-2.08	-2.39	1.15
NM_001114380	*ITGAL*	-2.08	-1.38	-1.50
NM_001168377	*KIAA0319*	-2.08	1.00	-2.08
NM_001012514	*ITM2C*	-2.08	-1.17	-1.77
NM_015198	*COBL*	-2.08	1.08	-2.24
NM_000043	*FAS*	-2.08	-2.77	1.33
NM_001127399	*YPEL5*	-2.08	-3.02	1.45
NR_045568	*IRF3*	-2.08	-2.77	1.33
NM_019555	*ARHGEF3*	-2.08	1.02	-2.13
NM_145715	*TIGD2*	-2.08	-1.91	-1.09
NM_002562	*P2RX7*	-2.09	-2.12	1.02
NM_001190202	*CES4A*	-2.09	-4.58	2.19
NM_001465	*FYB*	-2.09	-2.30	1.10
NM_001144382	*PLCL2*	-2.10	-1.87	-1.12
NR_048556	*TSN*	-2.11	-1.23	-1.71
NM_001102610	*TUBGCP5*	-2.11	-2.42	1.15
NM_001130720	*AHCYL2*	-2.11	-1.83	-1.15
NM_001258406	*IRG1*	-2.11	-6.18	2.93
NM_152305	*POGLUT1*	-2.11	-3.99	1.89
NM_020733	*HEG1*	-2.11	-1.89	-1.12
NM_001080	*ALDH5A1*	-2.12	1.19	-2.53
NM_001242900	*PPP6R2*	-2.12	-1.86	-1.14
NM_207370	*GPR153*	-2.12	-1.51	-1.41
NM_015537	*NELF*	-2.12	-1.96	-1.08
NR_027322	*LOC283070*	-2.12	-1.55	-1.37
NR_000025	*SNORD15B*	-2.13	-2.00	-1.06
NM_001126115	*TP53*	-2.13	-2.66	1.25
NM_012306	*FAIM2*	-2.14	2.56	-5.47
NM_001127396	*STXBP2*	-2.14	-1.15	-1.86
NM_001200029	*HYAL3*	-2.14	-2.67	1.25
NM_080283	*ABCA9*	-2.15	1.01	-2.16
NM_014974	*DIP2C*	-2.15	-1.11	-1.93

NM_001089	*ABCA3*	-2.15	-3.45	1.61
NM_001244249	*APEX1*	-2.15	-3.08	1.43
NM_018351	*FGD6*	-2.15	-2.38	1.11
NM_000820	*GAS6*	-2.16	-2.99	1.39
NM_001126054	*CASK*	-2.16	-1.69	-1.28
NR_073531	*STK25*	-2.16	-2.59	1.20
NM_021238	*FAM60A*	-2.16	-2.60	1.20
NM_002030	*FPR3*	-2.16	-2.14	-1.01
NR_024043	*LGALS9*	-2.17	-1.46	-1.48
NM_001242636	*IL31RA*	-2.17	-2.22	1.02
NM_000566	*FCGR1A*	-2.17	-2.38	1.10
NM_001048172	*MUTYH*	-2.19	-1.33	-1.64
NM_024592	*SRD5A3*	-2.19	-1.98	-1.11
NM_015136	*STAB1*	-2.19	-14.72	6.72
NM_014366	*GNL3*	-2.19	-2.69	1.23
NM_001206480	*ELMO1*	-2.19	-1.01	-2.17
NM_001244910	*FCGR1B*	-2.19	-2.84	1.29
NR_028301	*LOC344595*	-2.20	-2.42	1.10
NR_033951	*P2RX7*	-2.20	-1.97	-1.11
NM_181508	*HPS5*	-2.20	-1.65	-1.33
NM_001248003	*ARNTL2*	-2.20	-3.03	1.38
NM_001077594	*EXOC3L4*	-2.20	-2.03	-1.08
NR_047677	*TARS*	-2.21	-1.07	-2.07
NM_022468	*MMP25*	-2.21	-2.80	1.27
NM_006227	*PLTP*	-2.21	-2.07	-1.07
NM_194294	*IDO2*	-2.21	-1.94	-1.14
NM_001271581	*DNAJC10*	-2.22	-1.97	-1.12
NM_022845	*CBFB*	-2.22	-2.00	-1.11
NM_014435	*NAAA*	-2.22	-1.98	-1.12
NM_021175	*HAMP*	-2.22	-4.33	1.95
NM_001024808	*BCL7A*	-2.23	-3.26	1.46
NM_001271023	*ATRIP*	-2.23	-1.66	-1.35
NM_014890	*FILIP1L*	-2.24	-3.66	1.63
NM_145805	*ISL2*	-2.24	-1.46	-1.53
NM_145659	*IL27*	-2.25	-1.70	-1.32
NM_020440	*PTGFRN*	-2.25	-3.35	1.49
NM_015431	*TRIM58*	-2.25	-2.19	-1.03
NM_030664	*PTER*	-2.25	-1.82	-1.24
NM_001184718	*TIPARP*	-2.26	-2.71	1.20
NM_001206925	*CD86*	-2.27	-1.15	-1.96

NM_001242485	*EIF1AD*	-2.27	1.18	-2.69
NM_001207024	*M6PR*	-2.28	-1.73	-1.31
NM_001243228	*TCF4*	-2.28	-3.55	1.56
NM_030669	*PTPRO*	-2.28	-1.34	-1.70
NM_198156	*VHL*	-2.28	-1.50	-1.52
NM_033305	*VPS13A*	-2.29	-1.72	-1.33
NR_004399	*SNORD86*	-2.29	-1.71	-1.34
NM_020759	*STARD9*	-2.29	-2.48	1.08
NM_001199829	*HORMAD1*	-2.29	-1.85	-1.24
NM_003105	*SORL1*	-2.29	2.64	-6.04
NM_001201554	*ZNF821*	-2.29	-1.19	-1.93
NM_001146155	*PTGR2*	-2.30	-2.10	-1.09
NM_024111	*CHAC1*	-2.30	-1.92	-1.20
NM_004032	*DDO*	-2.30	-1.08	-2.14
NM_145279	*MOB3C*	-2.31	-1.96	-1.18
NM_002977	*SCN9A*	-2.31	-2.16	-1.07
NR_003320.1	*SNORD116-5*	-2.31	-2.34	1.01
NR_003322	*SNORD116-7*	-2.31	-2.34	1.01
NM_020397	*CAMK1D*	-2.31	-2.12	-1.09
NM_020477	*ANK1*	-2.31	-1.06	-2.18
NM_001199480	*ZNF193*	-2.32	-1.86	-1.24
NM_001172656	*ZFYVE28*	-2.32	-1.24	-1.86
NM_023001	*ARID4A*	-2.32	-2.14	-1.08
NM_012166	*FBXO10*	-2.33	-2.41	1.03
NM_203433	*PSMG1*	-2.33	1.01	-2.36
NM_015719	*COL5A3*	-2.33	-6.93	2.97
NM_003149	*STAC*	-2.33	2.33	-5.44
NM_001242921	*PLTP*	-2.34	-2.16	-1.08
NM_004883	*NRG2*	-2.34	-4.64	1.98
NM_023000	*ARID4A*	-2.35	-1.69	-1.40
NM_001172633	*OLR1*	-2.36	2.72	-6.43
NM_022140	*EPB41L4A*	-2.37	-2.02	-1.18
NM_002207	*ITGA9*	-2.37	-4.62	1.95
NM_001017986	*FCGR1B*	-2.38	-3.19	1.34
NM_177937	*GOLM1*	-2.39	-1.53	-1.56
NM_000014	*A2M*	-2.39	-1.60	-1.49
NM_152793	*C7orf41*	-2.40	-5.03	2.10
NM_001271711	*SLC2A8*	-2.40	-2.15	-1.12
NM_003456	*ZNF205*	-2.40	-1.66	-1.45
NR_033956	*P2RX7*	-2.42	-2.76	1.14

NM_001190810	*AGAP9*	-2.43	-1.70	-1.44
NM_002636	*PHF1*	-2.44	-1.08	-2.25
NM_173558	*FGD2*	-2.44	-2.46	1.01
NM_001001555	*GRB10*	-2.46	-3.36	1.37
NM_001928	*CFD*	-2.46	-2.85	1.16
NM_052998	*ADC*	-2.48	-1.46	-1.70
NM_139320	*CHRFAM7A*	-2.48	-2.56	1.03
NM_003649	*DDO*	-2.48	-2.36	-1.05
NM_001040453	*FAM63B*	-2.48	-1.35	-1.84
NM_001169107	*FAM21C*	-2.50	-1.78	-1.41
NM_001130831	*GAS7*	-2.50	-6.26	2.51
NM_203416	*CD163*	-2.51	-18.66	7.44
NM_001195790	*SLFN12L*	-2.51	1.06	-2.67
NM_001172632	*OLR1*	-2.51	3.46	-8.70
NM_016483	*PHF7*	-2.52	1.04	-2.61
NM_002336	*LRP6*	-2.52	-3.26	1.29
NM_016347	*NAT8B*	-2.53	-1.38	-1.83
NM_001166688	*PFKM*	-2.53	-2.62	1.04
NR_028500	*LDHA*	-2.54	-3.93	1.55
NM_001267584	*UBOX5*	-2.54	-3.41	1.34
NM_001243723	*SETMAR*	-2.55	-1.46	-1.74
NM_019857	*CTPS2*	-2.55	-2.65	1.04
NM_001167594	*CTSA*	-2.55	-1.24	-2.05
NM_145034	*TOR1AIP2*	-2.55	-3.90	1.53
NM_000573	*CR1*	-2.56	-7.14	2.79
NR_033243	*UBAP1*	-2.57	-1.03	-2.49
NM_032367	*ZBED3*	-2.57	-1.44	-1.79
NM_001258280	*ZNF501*	-2.57	-1.29	-1.99
NM_152872	*FAS*	-2.57	-2.67	1.04
NR_027484	*FCGR1C*	-2.57	-3.06	1.19
NM_014398	*LAMP3*	-2.57	-4.56	1.77
NM_018844	*BCAP29*	-2.57	-1.95	-1.32
NM_006744	*RBP4*	-2.58	-1.05	-2.45
NM_130439	*MXI1*	-2.59	-3.00	1.16
NM_006770	*MARCO*	-2.59	2.88	-7.49
NR_033738	*LOC440300*	-2.60	-2.43	-1.07
NM_001253845	*ADM2*	-2.60	-2.18	-1.19
NM_018441	*PECR*	-2.60	-2.00	-1.30
NR_033950	*P2RX7*	-2.61	-2.11	-1.24
NR_046324	*CHRNA7*	-2.61	-3.59	1.37

NM_001206974	*DHPS*	-2.62	-1.09	-2.41
NM_000072	*CD36*	-2.63	-6.01	2.29
NM_016577	*RAB6B*	-2.64	-1.44	-1.83
NM_001258040	*HARS*	-2.65	-1.99	-1.33
NM_001005374	*LRSAM1*	-2.67	-1.89	-1.41
NM_001001522	*TAGLN*	-2.67	-1.84	-1.45
NM_017688	*BSPRY*	-2.67	-2.48	-1.08
NM_001522	*GUCY2F*	-2.69	-2.26	-1.19
NM_001253823	*BLVRA*	-2.69	-4.00	1.49
NR_033955	*P2RX7*	-2.70	-2.97	1.10
NM_145267	*C6orf57*	-2.70	-2.06	-1.31
NM_012224	*NEK1*	-2.70	-3.08	1.14
NM_000210	*ITGA6*	-2.70	-2.09	-1.29
NM_199349	*KCP*	-2.72	-2.03	-1.34
NM_015196	*KIAA0922*	-2.73	-4.24	1.55
NM_001258249	*UTY*	-2.74	1.21	-3.33
NM_203386	*RNH1*	-2.75	-1.82	-1.51
NM_032515	*BOK*	-2.75	-2.42	-1.14
NR_002915	*SNORA74A*	-2.77	-3.63	1.31
NM_001256430	*STON2*	-2.77	-1.31	-2.12
NM_001003680	*LEPR*	-2.78	-1.39	-2.00
NM_001242640	*BRCC3*	-2.78	-3.39	1.22
NM_133436	*ASNS*	-2.78	-2.04	-1.36
NM_001003696	*ATP5J*	-2.81	-1.42	-1.97
NM_001242902	*AKAP1*	-2.83	-1.58	-1.79
NM_001166057	*PEPD*	-2.84	-2.61	-1.09
NM_001135176	*NEDD1*	-2.84	-2.68	-1.06
NM_001029955	*DCAF4L1*	-2.87	-2.06	-1.40
NM_005845	*ABCC4*	-2.88	-4.54	1.58
NM_001145657	*RAP1GAP*	-2.88	2.05	-5.90
NM_001033551	*TOM1L2*	-2.88	-1.14	-2.53
NM_005410	*SEPP1*	-2.89	-6.16	2.13
NR_045213	*FCGR1B*	-2.90	-3.63	1.25
NM_183005	*RPP38*	-2.90	-1.51	-1.92
NM_006207	*PDGFRL*	-2.91	-2.53	-1.15
NM_003711	*PPAP2A*	-2.91	-2.46	-1.18
NM_032438	*L3MBTL3*	-2.92	-2.29	-1.28
NM_001251888	*ASPSCR1*	-2.93	-1.45	-2.01
NM_000305	*PON2*	-2.93	-2.90	-1.01
NM_033104	*STON2*	-2.94	-1.21	-2.43

NM_014787	*DNAJC6*	-2.95	-1.83	-1.61
NM_003083	*SNAPC2*	-2.95	-1.38	-2.15
NM_007293.8	*C4A*	-2.96	-1.98	-1.49
NM_001002029.1	*C4B*	-2.96	-2.01	-1.47
NM_001242823.1	*LOC100293534*	-2.96	-2.01	-1.47
NM_152998	*EZH2*	-2.96	-4.14	1.40
NM_001014975	*CFH*	-2.96	-2.78	-1.07
NM_182676	*PLTP*	-2.97	-1.23	-2.43
NM_001164755	*ASPH*	-2.99	-4.65	1.55
NM_194436	*LDHD*	-3.00	-1.24	-2.42
NM_003039	*SLC2A5*	-3.00	-5.28	1.76
NM_033128	*SCIN*	-3.02	-1.39	-2.17
NM_001032282	*KLF10*	-3.03	-4.03	1.33
NM_001178008	*CBS*	-3.03	-2.19	-1.38
NM_001166356	*SHMT2*	-3.06	-1.65	-1.85
NM_021914	*CFL2*	-3.06	-1.45	-2.11
NM_194322	*OTOF*	-3.08	-1.13	-2.73
NM_002187	*IL12B*	-3.09	4.94	-15.28
NR_045555	*ENPP2*	-3.12	-4.77	1.53
NM_001145145	*SLC1A5*	-3.13	-1.51	-2.07
NM_001172746	*SEC23B*	-3.16	-1.46	-2.17
NM_174902	*LDLRAD3*	-3.17	-8.48	2.68
NM_001100428	*RAP1GDS1*	-3.18	-1.18	-2.70
NR_073097	*TRAPPC3*	-3.19	-1.03	-3.08
NM_182762	*MACC1*	-3.19	-1.23	-2.59
NM_001206897	*ALDH1A2*	-3.20	-1.16	-2.74
NM_138712	*PPARG*	-3.20	1.26	-4.04
NM_152536	*FGD5*	-3.21	1.56	-4.99
NM_002397	*MEF2C*	-3.24	-4.77	1.47
NR_033954	*P2RX7*	-3.24	-2.87	-1.13
NM_002873	*RAD17*	-3.24	-3.74	1.15
NM_001033025	*EXTL2*	-3.26	-4.50	1.38
NM_001193431	*PTPN22*	-3.28	-1.59	-2.06
NM_001439	*EXTL2*	-3.28	-3.90	1.19
NR_027752	*ALDH1L2*	-3.30	-3.23	-1.02
NM_138971	*BACE1*	-3.31	-2.92	-1.13
NR_045103	*RNF146*	-3.33	-2.64	-1.26
NM_201432	*GAS7*	-3.33	-6.07	1.82
NM_018848	*MKKS*	-3.34	-2.09	-1.60
NM_004235	*KLF4*	-3.36	-1.43	-2.36

NM_001766	*CD1D*	-3.37	-3.48	1.03
NM_001764	*CD1B*	-3.39	1.00	-3.39
NM_003088	*FSCN1*	-3.39	-11.54	3.40
NM_181526	*MYL9*	-3.43	-1.23	-2.80
NM_001258291	*PRSS36*	-3.44	-1.43	-2.41
NM_054012	*ASS1*	-3.44	-5.68	1.65
NM_001242768	*MTHFD1L*	-3.45	-3.71	1.08
NM_001174092	*TMEM185A*	-3.46	-2.23	-1.55
NM_001101801	*FAM13B*	-3.46	-3.88	1.12
NR_073037	*STOM*	-3.46	-3.22	-1.08
NR_038335	*CLU*	-3.48	-2.39	-1.46
NM_001033045	*GPR155*	-3.48	-1.26	-2.76
NM_001146008	*SLCO5A1*	-3.52	-12.01	3.41
NM_004244	*CD163*	-3.52	-25.98	7.38
NR_037582	*AP3S2*	-3.56	-1.83	-1.94
NM_004181	*UCHL1*	-3.57	-3.81	1.07
NM_006398.3	*UBD*	-3.57	-6.29	1.76
NM_014488	*RAB30*	-3.58	-3.39	-1.06
NM_001243752	*C7orf49*	-3.62	-2.11	-1.71
NM_001177507	*AOAH*	-3.62	-5.49	1.51
NM_198436	*AURKA*	-3.66	-1.13	-3.24
NM_001206984	*METTL23*	-3.68	-1.43	-2.58
NM_001976	*ENO3*	-3.69	-1.98	-1.87
NM_001243755	*C7orf49*	-3.70	-1.20	-3.08
NM_001025197	*CHI3L2*	-3.74	-3.70	-1.01
NM_001171158	*SIGLEC10*	-3.75	-2.77	-1.36
NM_001040092	*ENPP2*	-3.75	-10.79	2.88
NM_138790	*PLD4*	-3.76	-1.11	-3.39
NM_138972	*BACE1*	-3.77	-2.60	-1.45
NM_001256909	*CYBRD1*	-3.78	-4.39	1.16
NM_001271852	*CEP57L1*	-3.95	-1.30	-3.03
NM_001042402	*NAAA*	-3.96	-3.76	-1.05
NM_000071	*CBS*	-4.06	-2.69	-1.51
NM_001130859	*PPIP5K1*	-4.09	-4.11	1.01
NM_000857	*GUCY1B3*	-4.09	-6.51	1.59
NM_001127383	*CYBRD1*	-4.11	-3.05	-1.35
NM_152348	*WDR81*	-4.13	-1.13	-3.67
NM_001128933	*SYNPO2*	-4.14	-8.34	2.01
NM_001099	*ACPP*	-4.18	-3.59	-1.16
NM_016324	*ZNF274*	-4.22	-1.54	-2.74

NM_006623	*PHGDH*	-4.23	-3.63	-1.17
NM_016201	*AMOTL2*	-4.42	-7.11	1.61
NM_004447	*EPS8*	-4.46	-4.46	1.00
NM_001013398	*IGFBP3*	-4.52	-21.74	4.81
NM_015900	*PLA1A*	-4.52	-2.11	-2.14
NM_001127493	*ANK2*	-4.55	-3.69	-1.23
NM_001018056	*VLDLR*	-4.58	-5.66	1.23
NM_004384	*CSNK1G3*	-4.59	-3.93	-1.17
NM_001127242	*BBC3*	-4.65	-3.04	-1.53
NM_002852	*PTX3*	-4.73	-5.51	1.17
NR_037775	*MIA-RAB4B*	-4.81	-1.74	-2.76
NM_001001550	*GRB10*	-4.82	-4.60	-1.05
NM_001130687	*GUCY1A3*	-4.84	-6.32	1.30
NM_001128934	*SYNPO2*	-4.88	-7.28	1.49
NM_001148	*ANK2*	-4.92	-5.35	1.09
NM_001134194	*ACPP*	-4.93	-4.39	-1.12
NM_002416	*CXCL9*	-5.06	-5.15	1.02
NR_046480	*ROGDI*	-5.08	-1.09	-4.64
NM_021101	*CLDN1*	-5.08	-1.20	-4.22
NM_001130723	*AHCYL2*	-5.33	-12.04	2.26
NM_001142345	*CMKLR1*	-5.39	-1.92	-2.81
NM_001034173	*ALDH1L2*	-5.56	-4.98	-1.12
NM_000598	*IGFBP3*	-5.74	-30.36	5.29
NM_001006605	*FAM69A*	-5.89	-4.97	-1.18
NM_001130863	*ENPP2*	-6.30	-14.56	2.31
NM_001242948	*EPDR1*	-6.30	-3.37	-1.87
NM_001024845	*SLC6A9*	-6.39	-4.13	-1.55
NM_001221	*CAMK2D*	-6.68	-2.23	-3.00
NM_001130683	*GUCY1A3*	-7.04	-8.88	1.26
NM_021154	*PSAT1*	-8.17	-3.61	-2.27
NM_005409	*CXCL11*	-8.89	-7.88	-1.13
NM_001565	*CXCL10*	-12.64	-8.26	-1.53

Table S2. Transcripts that are uniquely and significantly differentially expressed in Mob-MDM(LPS/IFNγ) compared to both M-MDM(LPS/IFNγ) and GM-MDM(LPS/IFNγ).

		Fold Change		
**RefSeq transcript ID**	**Symbol**	**Mob-MDM(LPS/IFNγ) *vs.* GM-MDM(LPS/IFNγ)**	**Mob-MDM(LPS/IFNγ) *vs.* M-MDM(LPS/IFNγ)**	**M-MDM(LPS/IFNγ) *vs.* GM-MDM(LPS/IFNγ)**
**Cytokines and Chemokines**				
NM_000758	*CSF2*	38.14	47.90	-1.26
NM_000619	*IFNG*	34.43	22.54	1.53
NM_002994	*CXCL5*	27.00	18.59	1.45
NM_172219	*CSF3*	17.84	16.66	1.07
NR_046035	*CXCL1*	12.45	7.96	1.56
NR_033662	*CSF3*	10.17	13.61	-1.34
NM_019618	*IL36G*	9.73	10.75	-1.10
NM_000576	*IL1B*	9.52	10.43	-1.09
NM_001511	*CXCL1*	7.34	8.90	-1.21
NM_002090	*CXCL3*	6.26	3.17	1.97
NM_002089	*CXCL2*	4.51	3.87	1.16
NM_002984	*CCL4*	3.90	2.90	1.35
NM_020530	*OSM*	3.75	5.81	-1.55
NM_000639	*FASLG*	3.10	2.51	1.24
NM_002983	*CCL3*	2.97	3.31	-1.12
NM_001172128	*IL16*	2.60	3.46	-1.33
NM_001198624	*TNFSF13*	2.28	3.04	-1.33
NM_002416	*CXCL9*	-5.06	-5.15	1.02
NM_005409	*CXCL11*	-8.89	-7.88	-1.13
NM_001565	*CXCL10*	-12.64	-8.26	-1.53
**G-protein coupled receptors**				
NM_006564	*CXCR6*	6.93	7.04	-1.02
NM_013308	*GPR171*	4.15	3.84	1.08
NM_001008540	*CXCR4*	4.06	3.49	1.16
NM_001992	*F2R*	3.46	3.37	1.03
NM_001271749	*GPR77*	2.45	2.64	-1.08
NM_003467	*CXCR4*	2.31	2.06	1.12
NM_005508	*CCR4*	2.23	2.23	1.00
**Growth Factors**				
NM_004883	*NRG2*	-2.34	-4.64	1.98

**Ion Channels**				
NM_002561	*P2RX5*	8.27	10.28	-1.24
NM_001680	*FXYD2*	3.98	3.98	1.00
NM_001204519	*P2RX5*	3.81	2.89	1.32
NM_001177428	*TRPV4*	2.85	4.41	-1.54
NM_002224	*ITPR3*	2.48	2.57	-1.04
NM_002562	*P2RX7*	-2.09	-2.12	1.02
NM_002977	*SCN9A*	-2.31	-2.16	-1.07
NR_033956	*P2RX7*	-2.42	-2.76	1.14
NR_033950	*P2RX7*	-2.61	-2.11	-1.24
NR_033955	*P2RX7*	-2.70	-2.97	1.10
NR_033954	*P2RX7*	-3.24	-2.87	-1.13
**Kinases**				
NM_005356	*LCK*	7.92	7.92	1.00
NM_001042600	*MAP4K1*	6.57	5.59	1.17
NM_001079	*ZAP70*	5.65	5.02	1.12
NM_006257	*PRKCQ*	5.04	3.62	1.39
NM_032415	*CARD11*	4.32	4.84	-1.12
NM_000245	*MET*	3.93	5.07	-1.29
NM_004336	*BUB1*	3.65	4.44	-1.22
NM_005030	*PLK1*	3.39	2.90	1.17
NM_001786	*CDK1*	3.20	2.76	1.16
NM_016441	*CRIM1*	3.06	3.12	-1.02
NM_002082	*GRK6*	2.97	2.51	1.18
NM_001211	*BUB1B*	2.76	2.94	-1.06
NM_001161560	*TNIK*	2.73	2.27	1.20
NM_001161562	*TNIK*	2.53	2.10	1.21
NM_031966	*CCNB1*	2.38	2.87	-1.21
NM_004217	*AURKB*	2.36	2.54	-1.08
NM_006096	*NDRG1*	2.35	2.26	1.04
NM_001570	*IRAK2*	2.27	3.06	-1.35
NM_001274	*CHEK1*	2.11	2.19	-1.04
NM_004834	*MAP4K4*	2.05	2.57	-1.25
NR_073531	*STK25*	-2.16	-2.59	1.20
NM_020397	*CAMK1D*	-2.31	-2.12	-1.09
NM_001166688	*PFKM*	-2.53	-2.62	1.04
NM_001522	*GUCY2F*	-2.69	-2.26	-1.19
NM_012224	*NEK1*	-2.70	-3.08	1.14
NM_006207	*PDGFRL*	-2.91	-2.53	-1.15
NM_004384	*CSNK1G3*	-4.59	-3.93	-1.17

**Peptidases**				
NM_006144	*GZMA*	5.8	5.1	1.1
NM_033423	*GZMH*	5.2	3.5	1.5
NM_080722	*ADAMTS14*	3.7	2.2	1.7
NM_139155	*ADAMTS14*	3.7	2.2	1.7
NM_007287	*MME*	3.5	3.1	1.1
NM_001335	*CTSW*	2.6	3.5	-1.4
NM_022468	*MMP25*	-2.2	-2.8	1.3
NM_001928	*CFD*	-2.5	-2.9	1.2
NM_138971	*BACE1*	-3.3	-2.9	-1.1
NM_004181	*UCHL1*	-3.6	-3.8	1.1
NM_138972	*BACE1*	-3.8	-2.6	-1.5
NM_004447	*EPS8*	-4.5	-4.5	1.0
**Phosphatases**				
NM_001789	*CDC25A*	2.63	2.61	1.01
NM_014750	*DLGAP5*	2.25	2.25	1.00
NM_003711	*PPAP2A*	-2.91	-2.46	-1.18
NM_001130859	*PPIP5K1*	-4.09	-4.11	1.01
NM_001099	*ACPP*	-4.18	-3.59	-1.16
NM_001134194	*ACPP*	-4.93	-4.39	-1.12
**Other Enzymes**				
NM_000096	*CP*	21.10	19.66	1.07
NM_001034	*RRM2*	12.53	13.10	-1.05
NR_046371	*CP*	9.91	7.26	1.36
NM_001071	*TYMS*	8.53	5.47	1.56
NM_052838	*SEPT1*	7.35	5.06	1.45
NM_206925	*CA12*	5.83	4.34	1.34
NM_001144970	*NEDD4L*	5.25	3.34	1.57
NM_001243121	*PDE4A*	4.82	2.97	1.62
NM_001067	*TOP2A*	4.81	5.10	-1.06
NM_001218	*CA12*	4.59	4.68	-1.02
NM_002263.1	*KIFC1*	4.55	4.61	-1.01
NM_002875	*RAD51*	3.92	2.97	1.32
NM_001165931	*RRM2*	3.48	3.48	1.00
NM_005916	*MCM7*	3.43	2.91	1.18
NM_001197221	*PDE4D*	3.25	3.90	-1.20
NM_014875	*KIF14*	2.90	2.90	1.00
NM_004130	*GYG1*	2.80	3.00	-1.07
NM_006907	*PYCR1*	2.77	2.76	1.01
NM_017640	*LRRC16A*	2.69	2.40	1.12

NM_003579	*RAD54L*	2.65	2.64	1.00
NM_012168	*FBXO2*	2.60	2.60	1.00
NM_001017420	*ESCO2*	2.46	2.29	1.08
NM_001017917	*CYB561*	2.45	2.34	1.04
NM_021615	*CHST6*	2.39	2.61	-1.09
NM_002528	*NTHL1*	2.03	2.45	-1.21
NM_001185074	*ZCCHC6*	2.02	2.53	-1.25
NM_015723	*PNPLA8*	2.01	2.90	-1.45
NM_020142	*NDUFA4L2*	-2.01	-2.10	1.05
NM_152305	*POGLUT1*	-2.11	-3.99	1.89
NM_001200029	*HYAL3*	-2.14	-2.67	1.25
NM_006227	*PLTP*	-2.21	-2.07	-1.07
NM_001184718	*TIPARP*	-2.26	-2.71	1.20
NM_001146155	*PTGR2*	-2.30	-2.10	-1.09
NM_012166	*FBXO10*	-2.33	-2.41	1.03
NM_001242921	*PLTP*	-2.34	-2.16	-1.08
NM_003649	*DDO*	-2.48	-2.36	-1.05
NR_028500	*LDHA*	-2.54	-3.93	1.55
NM_001267584	*UBOX5*	-2.54	-3.41	1.34
NM_019857	*CTPS2*	-2.55	-2.65	1.04
NM_018441	*PECR*	-2.60	-2.00	-1.30
NM_001253823	*BLVRA*	-2.69	-4.00	1.49
NM_001242640	*BRCC3*	-2.78	-3.39	1.22
NM_133436	*ASNS*	-2.78	-2.04	-1.36
NM_000305	*PON2*	-2.93	-2.90	-1.01
NM_001164755	*ASPH*	-2.99	-4.65	1.55
NM_001178008	*CBS*	-3.03	-2.19	-1.38
NR_045555	*ENPP2*	-3.12	-4.77	1.53
NM_002873	*RAD17*	-3.24	-3.74	1.15
NM_001033025	*EXTL2*	-3.26	-4.50	1.38
NM_001439	*EXTL2*	-3.28	-3.90	1.19
NR_027752	*ALDH1L2*	-3.30	-3.23	-1.02
NR_045103	*RNF146*	-3.33	-2.64	-1.26
NM_054012	*ASS1*	-3.44	-5.68	1.65
NM_001242768	*MTHFD1L*	-3.45	-3.71	1.08
NM_014488	*RAB30*	-3.58	-3.39	-1.06
NM_001177507	*AOAH*	-3.62	-5.49	1.51
NM_001025197	*CHI3L2*	-3.74	-3.70	-1.01
NM_001256909	*CYBRD1*	-3.78	-4.39	1.16
NM_001042402	*NAAA*	-3.96	-3.76	-1.05

NM_000071	*CBS*	-4.06	-2.69	-1.51
NM_000857	*GUCY1B3*	-4.09	-6.51	1.59
NM_006623	*PHGDH*	-4.23	-3.63	-1.17
NM_001130687	*GUCY1A3*	-4.84	-6.32	1.30
NM_001034173	*ALDH1L2*	-5.56	-4.98	-1.12
N22:77M_001130683	*GUCY1A3*	-7.04	-8.88	1.26
**Transcription Regulators**				
NM_030915	*LBH*	8.14	5.32	1.53
NM_005442	*EOMES*	7.25	4.92	1.48
NM_002466	*MYBL2*	7.19	6.33	1.13
NM_004430	*EGR3*	5.59	4.98	1.12
NM_001131010	*SATB1*	5.50	4.42	1.25
NM_004091	*E2F2*	4.86	4.72	1.03
NM_005225	*E2F1*	3.79	5.86	-1.55
NM_002467	*MYC*	3.78	2.84	1.33
NM_012481	*IKZF3*	3.50	3.03	1.16
NM_001257410	*IKZF3*	3.32	2.66	1.24
NM_024007	*EBF1*	3.25	3.01	1.08
NM_001262	*CDKN2C*	3.23	2.54	1.27
NM_012258	*HEY1*	3.14	3.91	-1.25
NM_013282	*UHRF1*	3.07	2.71	1.13
NM_021953	*FOXM1*	2.78	3.54	-1.28
NM_022898	*BCL11B*	2.52	2.30	1.10
NM_173091	*NFATC2*	2.51	2.51	1.00
NM_004936	*CDKN2B*	2.42	2.07	1.17
NM_001202514	*MXD1*	2.42	2.66	-1.10
NM_199185	*NPM1*	2.39	2.18	1.10
NM_001170794	*BACH2*	2.23	3.56	-1.59
NM_002357	*MXD1*	2.12	2.43	-1.14
NM_015517	*HINFP*	-2.03	-2.22	1.09
NR_045568	*IRF3*	-2.08	-2.77	1.33
NM_001126115	*TP53*	-2.13	-2.66	1.25
NM_001248003	*ARNTL2*	-2.20	-3.03	1.38
NM_001243228	*TCF4*	-2.28	-3.55	1.56
NM_023001	*ARID4A*	-2.32	-2.14	-1.08
NM_130439	*MXI1*	-2.59	-3.00	1.16
NM_152998	*EZH2*	-2.96	-4.14	1.40
NM_001032282	*KLF10*	-3.03	-4.03	1.33
NM_002397	*MEF2C*	-3.24	-4.77	1.47
NM_201432	*GAS7*	-3.33	-6.07	1.82

**Transmembrane Receptors**				
NM_001767	*CD2*	14.85	11.00	1.35
NM_000733	*CD3E*	8.18	7.71	1.06
NM_012092	*ICOS*	7.04	6.73	1.04
NM_000655	*SELL*	6.12	5.45	1.12
NM_006725	*CD6*	5.79	5.46	1.06
NM_001559	*IL12RB2*	5.00	5.00	1.00
NM_003853	*IL18RAP*	4.92	4.44	1.11
NM_000734	*CD247*	4.56	5.37	-1.18
NM_000732	*CD3D*	4.20	4.20	1.00
NM_001258216	*IL12RB2*	3.72	3.21	1.16
NM_014207	*CD5*	3.32	2.86	1.16
NM_001099439	*EPHA10*	3.04	3.03	1.00
NM_000073	*CD3G*	2.77	2.77	1.00
NM_003855	*IL18R1*	2.65	2.57	1.03
NR_028076	*SCARF1*	2.22	2.17	1.02
NM_000043	*FAS*	-2.08	-2.77	1.33
NM_001242636	*IL31RA*	-2.17	-2.22	1.02
NM_000566	*FCGR1A*	-2.17	-2.38	1.10
NM_001244910	*FCGR1B*	-2.19	-2.84	1.29
NM_001017986	*FCGR1B*	-2.38	-3.19	1.34
NM_002336	*LRP6*	-2.52	-3.26	1.29
NM_152872	*FAS*	-2.57	-2.67	1.04
NR_027484	*FCGR1C*	-2.57	-3.06	1.19
NR_046324	*CHRNA7*	-2.61	-3.59	1.37
NM_000210	*ITGA6*	-2.70	-2.09	-1.29
NR_045213	*FCGR1B*	-2.90	-3.63	1.25
**Ligand-dependent nuclear receptors**				
NM_001202233	*NR4A1*	3.44	4.16	-1.21
NM_006186	*NR4A2*	2.71	3.16	-1.16
NM_134262	*RORA*	2.63	2.69	-1.02
NM_001042728	*RARG*	2.60	3.05	-1.17
**Transporters**				
NM_003645	*SLC27A2*	6.39	5.92	1.08
NM_004603	*STX1A*	3.92	2.47	1.59
NM_005733	*KIF20A*	3.56	3.44	1.04
NM_001136232	*SEC13*	3.46	2.50	1.38
NM_000228	*LAMB3*	2.99	2.45	1.22
NM_014331	*SLC7A11*	2.91	2.67	1.09

NR_024273	*SEC13*	2.88	2.65	1.09
NM_178859	*SLC51B*	2.45	3.48	-1.42
NM_003786	*ABCC3*	2.01	2.80	-1.39
NM_024490	*ATP10A*	-2.01	-2.19	1.09
NM_001112802	*SLC8A1*	-2.02	-2.48	1.23
NM_001089	*ABCA3*	-2.15	-3.45	1.61
NM_001271711	*SLC2A8*	-2.40	-2.15	-1.12
NM_005845	*ABCC4*	-2.88	-4.54	1.58
NM_003039	*SLC2A5*	-3.00	-5.28	1.76
NM_001018056	*VLDLR*	-4.58	-5.66	1.23
NM_001024845	*SLC6A9*	-6.39	-4.13	-1.55
**Others**				
NM_003537	*HIST1H3B*	16.97	14.05	1.21
NM_003531	*HIST1H3C*	16.43	11.00	1.49
NM_003534	*HIST1H3G*	15.88	11.89	1.34
NM_005322	*HIST1H1B*	13.48	6.97	1.93
NM_001005464.1	*HIST2H3A*	13.48	8.08	1.67
NM_021059.1	*HIST2H3C*	13.48	8.08	1.67
NM_021066	*HIST1H2AJ*	12.50	8.81	1.42
NM_003521	*HIST1H2BM*	12.44	9.80	1.27
NM_003511	*HIST1H2AL*	10.45	6.26	1.67
NM_021018	*HIST1H3F*	10.00	6.18	1.62
NM_003535	*HIST1H3J*	8.74	7.73	1.13
NM_001161443	*SH2D2A*	8.23	6.96	1.18
NM_024508	*ZBED2*	7.81	5.77	1.35
NM_052916	*RNF157*	7.76	7.44	1.04
NM_001005464	*HIST2H3A*	7.70	7.51	1.03
NM_021059	*HIST2H3C*	7.70	7.51	1.03
NM_001083116	*PRF1*	7.69	5.24	1.47
NM_001145966	*MKI67*	7.50	5.53	1.36
NM_017709	*FAM46C*	6.61	6.23	1.06
NM_005816	*CD96*	6.60	4.17	1.58
NM_006433	*GNLY*	6.12	4.85	1.26
NM_175055	*HIST3H2BB*	5.85	3.76	1.56
NM_021064	*HIST1H2AG*	5.78	2.96	1.95
NM_018410	*HJURP*	5.72	5.72	1.00
NM_003514	*HIST1H2AM*	5.70	2.87	1.99
NM_003536	*HIST1H3H*	5.58	3.07	1.82
NM_016448	*DTL*	5.55	5.52	1.01
NM_003548	*HIST2H4A*	5.32	3.15	1.69

NM_001034077	*HIST2H4B*	5.32	3.15	1.69
NM_003513	*HIST1H2AB*	5.28	3.73	1.42
NM_014220	*TM4SF1*	5.24	4.60	1.14
NM_003527	*HIST1H2BO*	5.14	3.00	1.71
NM_018136	*ASPM*	5.07	5.85	-1.15
NM_001123375	*HIST2H3D*	5.02	3.18	1.58
NM_133272	*FCAR*	4.87	4.42	1.10
NM_178844	*NLRC3*	4.86	3.69	1.32
NM_002000	*FCAR*	4.77	4.41	1.08
NM_018004	*TMEM45A*	4.71	3.23	1.46
NM_006137	*CD7*	4.65	3.05	1.52
NM_003548.1	*HIST2H4A*	4.50	2.84	1.59
NM_001034077.1	*HIST2H4B*	4.50	2.84	1.59
NM_201538	*NDRG2*	4.47	4.47	1.00
NM_016343	*CENPF*	4.42	3.12	1.42
NM_021058	*HIST1H2BJ*	4.39	2.35	1.87
NM_001168	*BIRC5*	4.37	4.30	1.02
NM_003546	*HIST1H4L*	4.36	3.42	1.28
NM_001164114	*CASS4*	4.36	4.68	-1.07
NM_002575	*SERPINB2*	4.30	4.57	-1.06
NM_003539	*HIST1H4D*	4.24	2.88	1.47
NM_012112	*TPX2*	4.19	4.78	-1.14
NM_003538	*HIST1H4A*	4.05	3.66	1.11
NM_002928	*RGS16*	4.05	2.14	1.89
NM_020356	*CASS4*	4.02	3.41	1.18
NM_001255	*CDC20*	3.99	3.44	1.16
NM_003523	*HIST1H2BE*	3.97	2.46	1.62
NM_003650	*CST7*	3.86	2.01	1.92
NM_004523	*KIF11*	3.85	3.87	-1.00
NM_003524	*HIST1H2BH*	3.77	3.07	1.23
NM_016426	*GTSE1*	3.72	3.72	1.00
NM_001003675	*LDLRAD4*	3.70	2.79	1.33
NM_018349	*MCTP2*	3.66	3.09	1.18
NM_012483	*GNLY*	3.60	2.73	1.32
NM_003525	*HIST1H2BI*	3.55	2.37	1.50
NM_001163524	*ITPRIPL1*	3.50	2.70	1.30
NM_182914	*SYNE2*	3.47	3.87	-1.12
NM_152515	*CKAP2L*	3.43	3.43	1.00
NM_001145664	*RFX8*	3.43	3.43	1.00
NM_030754	*SAA2*	3.37	2.54	1.33

NM_001237	*CCNA2*	3.33	2.78	1.20
NM_001254	*CDC6*	3.26	3.53	-1.08
NM_017720	*STAP2*	3.25	2.21	1.47
NM_001244950	*SPOCK2*	3.25	4.77	-1.47
NM_001079907	*ZNF331*	3.25	2.61	1.24
NM_032108	*SEMA6B*	3.24	3.50	-1.08
NM_144720	*JAKMIP1*	3.16	3.16	1.00
NM_003975	*SH2D2A*	3.15	3.15	1.00
NR_038380	*SLC7A11-AS1*	3.14	3.63	-1.15
NM_001144894	*CD209*	3.14	2.98	1.05
NM_003522	*HIST1H2BF*	3.14	2.49	1.26
NM_003544	*HIST1H4B*	3.12	2.57	1.22
NM_000608	*ORM2*	3.02	3.17	-1.05
NM_001264573	*KIF18B*	3.00	2.87	1.05
NM_022770	*GINS3*	2.99	2.85	1.05
NM_003542	*HIST1H4C*	2.94	2.19	1.34
NM_001252102	*KIF21B*	2.89	3.15	-1.09
NR_073484	*CDK2AP2*	2.79	2.25	1.24
NM_012310	*KIF4A*	2.77	2.77	1.00
NM_080881	*DBN1*	2.75	2.51	1.10
NM_003530	*HIST1H3D*	2.70	2.13	1.27
NM_000094	*COL7A1*	2.67	3.06	-1.15
NM_001242416	*WDR20*	2.65	2.44	1.09
NM_001017995	*SH3PXD2B*	2.65	2.02	1.31
NM_002358	*MAD2L1*	2.60	2.82	-1.08
NM_004867	*ITM2A*	2.58	2.58	1.00
NM_001242608	*NCAM1*	2.57	4.47	-1.74
NM_006101	*NDC80*	2.56	2.86	-1.12
NM_173799	*TIGIT*	2.55	2.73	-1.07
NM_138371	*PCED1B*	2.51	2.07	1.21
NM_013372	*GREM1*	2.47	2.41	1.02
NM_001145199	*C12orf75*	2.42	2.42	1.00
NR_028308	*BRE-AS1*	2.41	2.50	-1.04
NM_080616	*C20orf112*	2.41	2.58	-1.07
NM_018131	*CEP55*	2.38	2.80	-1.18
NM_018518	*MCM10*	2.37	2.37	1.00
NM_005338	*HIP1*	2.36	4.00	-1.70
NM_014450	*SIT1*	2.36	2.36	1.00
NM_198947	*FAM111B*	2.35	2.35	1.00
NM_201536	*NDRG2*	2.34	2.34	1.00

NM_182751	*MCM10*	2.32	2.30	1.01
NM_201539	*NDRG2*	2.31	2.05	1.13
NR_002720	*TRPC2*	2.28	2.61	-1.14
NM_001159531	*BEGAIN*	2.22	2.06	1.08
NR_045006	*NRON*	2.17	2.53	-1.17
NM_001145319	*PLS1*	2.16	2.81	-1.31
NM_133279	*FCAR*	2.15	2.15	1.00
NM_001761	*CCNF*	2.14	2.57	-1.20
NM_001009936	*PHF19*	2.10	2.10	1.00
NM_173564	*NYAP1*	2.06	2.06	1.00
NM_014448	*ARHGEF16*	2.03	2.15	-1.06
NM_004186	*SEMA3F*	2.03	2.03	1.00
NR_073090	*MEAF6*	2.02	2.60	-1.29
NM_152400	*C4orf32*	-2.00	-2.01	1.00
NR_003323	*SNORD116-8*	-2.01	-2.28	1.14
NM_001256394	*NOL8*	-2.01	-2.02	1.00
NM_015548	*DST*	-2.01	-2.85	1.41
NM_001025235	*TSPAN4*	-2.02	-3.62	1.79
NR_003337	*SNORD116-23*	-2.03	-2.64	1.30
NM_018649	*H2AFY2*	-2.04	-2.44	1.20
NM_201612	*IKBIP*	-2.05	-3.20	1.56
NM_024296	*CCDC28B*	-2.05	-2.17	1.06
NM_001207018	*SMTN*	-2.06	-2.93	1.42
NR_003340	*SNORD116-26*	-2.07	-2.31	1.12
NM_017549	*EPDR1*	-2.07	-2.95	1.42
NM_001130709	*RUFY3*	-2.07	-3.57	1.72
NM_001144936	*C11orf95*	-2.08	-2.39	1.15
NM_001127399	*YPEL5*	-2.08	-3.02	1.45
NM_001465	*FYB*	-2.09	-2.30	1.10
NM_001102610	*TUBGCP5*	-2.11	-2.42	1.15
NM_018351	*FGD6*	-2.15	-2.38	1.11
NM_021238	*FAM60A*	-2.16	-2.60	1.20
NM_002030	*FPR3*	-2.16	-2.14	-1.01
NM_014366	*GNL3*	-2.19	-2.69	1.23
NR_028301	*LOC344595*	-2.20	-2.42	1.10
NM_001077594	*EXOC3L4*	-2.20	-2.03	-1.08
NM_021175	*HAMP*	-2.22	-4.33	1.95
NM_001024808	*BCL7A*	-2.23	-3.26	1.46
NM_014890	*FILIP1L*	-2.24	-3.66	1.63
NM_020440	*PTGFRN*	-2.25	-3.35	1.49

NM_015431	*TRIM58*	-2.25	-2.19	-1.03
NM_020759	*STARD9*	-2.29	-2.48	1.08
NR_003320.1	*SNORD116-5*	-2.31	-2.34	1.01
NR_003322	*SNORD116-7*	-2.31	-2.34	1.01
NM_022140	*EPB41L4A*	-2.37	-2.02	-1.18
NM_002207	*ITGA9*	-2.37	-4.62	1.95
NM_173558	*FGD2*	-2.44	-2.46	1.01
NM_001001555	*GRB10*	-2.46	-3.36	1.37
NM_139320	*CHRFAM7A*	-2.48	-2.56	1.03
NM_145034	*TOR1AIP2*	-2.55	-3.90	1.53
NM_014398	*LAMP3*	-2.57	-4.56	1.77
NR_033738	*LOC440300*	-2.60	-2.43	-1.07
NM_001253845	*ADM2*	-2.60	-2.18	-1.19
NM_017688	*BSPRY*	-2.67	-2.48	-1.08
NM_145267	*C6orf57*	-2.70	-2.06	-1.31
NM_199349	*KCP*	-2.72	-2.03	-1.34
NM_015196	*KIAA0922*	-2.73	-4.24	1.55
NM_032515	*BOK*	-2.75	-2.42	-1.14
NR_002915	*SNORA74A*	-2.77	-3.63	1.31
NM_001135176	*NEDD1*	-2.84	-2.68	-1.06
NM_001029955	*DCAF4L1*	-2.87	-2.06	-1.40
NM_032438	*L3MBTL3*	-2.92	-2.29	-1.28
NM_001002029.1	*C4B*	-2.96	-2.01	-1.47
NM_001242823.1	*LOC100293534*	-2.96	-2.01	-1.47
NM_001014975	*CFH*	-2.96	-2.78	-1.07
NM_001766	*CD1D*	-3.37	-3.48	1.03
NM_001101801	*FAM13B*	-3.46	-3.88	1.12
NR_073037	*STOM*	-3.46	-3.22	-1.08
NR_038335	*CLU*	-3.48	-2.39	-1.46
NM_006398.3	*UBD*	-3.57	-6.29	1.76
NM_001243752	*C7orf49*	-3.62	-2.11	-1.71
NM_001171158	*SIGLEC10*	-3.75	-2.77	-1.36
NM_016201	*AMOTL2*	-4.42	-7.11	1.61
NM_001127493	*ANK2*	-4.55	-3.69	-1.23
NM_001127242	*BBC3*	-4.65	-3.04	-1.53
NM_002852	*PTX3*	-4.73	-5.51	1.17
NM_001001550	*GRB10*	-4.82	-4.60	-1.05
NM_001128934	*SYNPO2*	-4.88	-7.28	1.49
NM_001148	*ANK2*	-4.92	-5.35	1.09
NM_001006605	*FAM69A*	-5.89	-4.97	-1.18

Table S3. Predicted upstream regulators of transcripts that are uniquely and significantly modulated in Mob-MDM(LPS/IFNγ) relative to M-MDM(LPS/IFNγ) and GM-MDM-(LPS/IFNγ).

	Activation z-score	
**Upstream regulators**	**Mob-MDM(LPS/IFNγ)** *vs.* **GM-MDM(LPS/IFNγ) and M-MDM(LPS/IFNγ)**	**M-MDM(LPS/IFNγ)** *vs.* **GM-MDM(LPS/IFNγ)**
CSF2	4.61	-0.64
IL2	3.98	1.92
FOXM1	3.84	0.73
AREG	3.77	0.90
PTGER2	3.75	0.60
RABL6	3.75	-0.60
ERK1/2	3.66	1.73
CCND1	3.60	1.47
IL15	3.57	1.75
MITF	3.54	-1.27
E2F3	3.47	0.96
MIF	3.39	0.10
MAPK1	3.36	0.19
CEBPB	3.22	1.00
HGF	3.20	-1.05
E. coli B5 lipopolysaccharide	3.19	0.42
KITLG	3.05	0.88
MED1	3.03	-0.43
IL17A	3.02	1.06
EGF	2.99	0.17
Vegf	2.96	0.75
JUN	2.93	-0.22
RAF1	2.90	-0.22
IL1	2.88	0.54
ERBB2	2.86	0.41
FGF2	2.85	1.19
TBX2	2.84	-0.26
EGFR	2.80	0.77
TCR	2.80	1.32
IL17a dimer	2.79	-0.19
estrogen	2.77	1.24
CCL5	2.74	0.94
CSF1	2.73	1.09
leukotriene D4	2.62	0.35
MET	2.62	0.31
Hsp90	2.62	1.50
LEP	2.61	-1.21
MAPK7	2.61	-1.07

tributyrin	2.61	0.80
NTRK2	2.61	1.12
ICAM1	2.60	1.78
Fcer1	2.60	-0.86
Pam3-Cys-Ser-Lys4	2.60	0.49
IRF2	2.59	1.10
peptidoglycan	2.58	0.26
TNFSF11	2.58	1.51
Salmonella enterica serotype abortus equi lipopolysaccharide	2.53	0.93
lipopolysaccharide	2.52	0.34
BCR (complex)	2.52	1.01
ionomycin	2.45	0.05
1,4-bis[2-(3,5-dichloropyridyloxy)] benzene	2.45	-0.31
SELPLG	2.45	0.45
E2F1	2.44	1.75
ICOS	2.43	1.71
IL-17f dimer	2.43	-0.97
MAP2K1	2.42	1.50
POMC	2.42	0.06
TRAF6	2.41	0.19
zymosan	2.41	0.20
histamine	2.41	-0.84
CD86	2.40	1.72
TLR5	2.40	0.34
EOMES	2.40	0.95
Ap1	2.40	1.06
hemozoin	2.39	0.97
PGF	2.36	1.30
E2F2	2.34	1.72
HRAS	2.32	0.36
PDGF BB	2.31	1.32
CYR61	2.29	0.61
dextran sulfate	2.29	0.16
IL1B	2.29	1.71
metribolone	2.27	0.29
CREB1	2.27	0.75
enterotoxin B	2.25	0.71
ASCL1	2.24	-0.45
EWSR1	2.24	0.00
FSH	2.24	0.45
lipoarabinomannan	2.24	0.45
SMOC2	2.24	0.45
CD40	2.23	1.30

IL12 (complex)	2.22	1.21
A23187	2.22	-0.33
IL3	2.21	-0.29
AIMP1	2.21	0.39
IGF1	2.20	-0.44
Mapk	2.20	1.45
TREM1	2.19	-0.04
MALP-2s	2.19	1.46
5-hydroxytryptamine	2.19	-0.71
TNFSF13B	2.19	0.62
RET	2.18	-0.32
MYD88	2.18	0.02
F2RL1	2.18	-0.13
ITK	2.17	0.29
NR4A1	2.17	0.65
MYB	2.15	1.11
mycophenolic acid	2.12	0.00
salmonella minnesota R595 lipopolysaccharides	2.11	0.64
Ccl2	2.11	0.59
uric acid	2.10	-0.20
TAC1	2.10	0.49
E2f	2.10	-0.35
phytohemagglutinin	2.09	0.69
8-bromo-cAMP	2.09	-0.16
phorbol myristate acetate	2.08	-0.29
resiquimod	2.08	-0.03
cholesterol	2.06	0.17
IL17F	2.06	1.28
26s Proteasome	2.03	-0.03
E. coli lipopolysaccharide	2.01	0.29
LDL	2.01	0.86
poly rI:rC-RNA	2.01	1.00
YAP1	2.00	-1.00
LATS2	2.00	-1.00
ANKRD42	2.00	-1.00
glucagon	2.00	0.00
GNAI3	2.00	0.00
4-nitroquinoline-1-oxide	2.00	0.00
Fibrinogen	2.00	1.00
CARM1	2.00	1.00
12-(3-adamantan-1-yl-ureido) dodecanoic acid	-2.00	-1.00
WWTR1	-2.00	-1.00
SMARCE1	-2.00	-1.00
vitamin D	-2.00	-1.00

TAF4	-2.00	0.00
panobinostat	-2.00	0.00
MSTN	-2.00	0.00
ATF3	-2.06	-0.94
resveratrol	-2.06	1.84
CTLA4	-2.11	-1.40
alvocidib	-2.13	0.25
Irgm1	-2.16	-0.28
NCOA2	-2.17	-0.08
Ifn	-2.17	0.27
silibinin	-2.17	-0.21
VIP	-2.17	-1.25
TSC22D3	-2.18	-0.29
ABCG1	-2.19	-0.18
2-deoxyglucose	-2.20	-0.44
methotrexate	-2.21	0.70
triflusal	-2.21	0.39
SMARCB1	-2.22	0.43
CBX5	-2.22	-1.11
Ciap	-2.22	0.55
UXT	-2.23	1.06
DYRK1A	-2.24	0.45
BMS-690514	-2.24	-1.34
celecoxib	-2.24	0.24
TP73	-2.33	-1.53
dexamethasone	-2.39	-0.98
glucocorticoid	-2.39	1.21
sulforafan	-2.39	0.80
PRDM1	-2.40	-1.17
mir-21	-2.41	-0.70
salicylic acid	-2.42	0.59
Go 6976	-2.43	-0.13
rotenone	-2.43	-0.13
fulvestrant	-2.50	-0.44
zinc	-2.56	0.97
medroxyprogesterone acetate	-2.57	0.73
simvastatin	-2.58	0.05
infliximab	-2.61	-0.69
2-amino-5-phosphonovaleric acid	-2.63	-0.24
Rb	-2.65	-0.94
CDKN2A	-2.65	-1.76
imipramine blue	-2.75	-0.57
KDM5B	-2.81	1.87
alefacept	-2.81	-1.91
epigallocatechin-gallate	-2.90	-0.88

U0126	-3.05	-0.80
vorinostat	-3.05	-0.92
aspirin	-3.08	-0.63
NUPR1	-3.15	-1.96
RBL2	-3.18	-0.28
let-7	-3.20	0.02
RBL1	-3.23	-1.59
miR-155-5p (miRNAs w/seed UAAUGCU)	-3.27	-0.82
curcumin	-3.27	0.17
TP53	-3.29	1.21
RB1	-3.35	-0.69
calcitriol	-3.49	-0.43
CD28	-3.52	-0.11
CDKN1A	-3.66	0.04
CD3	-3.97	-1.86
LY294002	-4.31	-1.74

Table S4. Predicted biological functions that are uniquely and significantly modulated in Mob-MDM(LPS/IFNγ) relative to M-MDM(LPS/IFNγ) and GM-MDM-(LPS/IFNγ).

	Activation z-score	
**Diseases and Bio Functions**	**Mob-MDM(LPS/IFNγ)** *vs.* **GM-MDM(LPS/IFNγ) and M-MDM(LPS/IFNγ)**	**M-MDM(LPS/IFNγ)** *vs.* **GM-MDM(LPS/IFNγ)**
Activation of blood cells	3.70	1.05
Activation of cells	3.63	1.08
Cellular homeostasis	3.62	0.55
Activation of lymphatic system cells	3.60	1.44
Activation of leukocytes	3.60	0.88
Leukopoiesis	3.57	0.37
Cell cycle progression	3.51	-0.66
Activation of lymphocytes	3.50	1.33
Differentiation of mononuclear leukocytes	3.45	-0.12
Cell proliferation of tumor cell lines	3.34	0.68
Lymphocyte homeostasis	3.33	0.47
Hematopoiesis of mononuclear leukocytes	3.33	0.07
Growth of malignant tumor	3.33	-0.74
Cell survival	3.31	0.30
Lymphopoiesis	3.28	0.24
Stimulation of cells	3.23	0.81
Mitosis	3.20	-1.47
Metabolism of DNA	3.14	0.85
Infiltration by myeloid cells	3.12	1.19
Cellular infiltration by myeloid cells	3.03	1.06
T cell development	2.97	0.07
Proliferation of tumor cells	2.96	-0.63
Activation of neutrophils	2.95	0.38
Cellular infiltration by granulocytes	2.90	0.91
Activation of T lymphocytes	2.88	1.38
Synthesis of reactive oxygen species	2.87	0.26
Cell viability	2.87	-0.12
Proliferation of cancer cells	2.85	-0.04

Metabolism of reactive oxygen species	2.84	0.28
Recruitment of cells	2.83	0.03
Activation of granulocytes	2.83	0.34
Cell movement of myeloid cells	2.80	1.13
Maturation of leukocytes	2.80	-0.67
Growth of epithelial tissue	2.79	-0.74
Mobilization of neutrophils	2.77	-0.19
Cellular infiltration by phagocytes	2.74	0.84
Cell proliferation of carcinoma cell lines	2.70	-0.57
Differentiation of helper T lymphocytes	2.65	-1.15
Angiogenesis	2.58	0.13
Development of vasculature	2.58	0.13
Chemotaxis of leukocytes	2.55	1.62
Mobilization of leukocytes	2.54	0.31
Influx of leukocytes	2.54	-0.21
Chemotaxis of myeloid cells	2.54	1.25
Binding of myeloid cells	2.52	1.22
Ion homeostasis of cells	2.51	0.94
Differentiation of T lymphocytes	2.50	0.54
Mobilization of myeloid cells	2.49	-0.54
Stimulation of leukocytes	2.49	0.54
Interaction of DNA	2.47	1.57
Chemotaxis	2.42	0.84
Flux of Ca2+	2.41	0.96
Maturation of lymphatic system cells	2.37	-0.21
Adhesion of immune cells	2.35	1.14
Accumulation of cells	2.34	-0.87
Mobilization of blood cells	2.33	0.08
Homing of cells	2.32	0.89
Stimulation of lymphocytes	2.31	1.18
Binding of professional phagocytic cells	2.31	1.12
Flux of ion	2.30	0.63
Cell movement of granulocytes	2.28	0.67
Tumorigenesis of neuroepithelial tumor	2.27	-0.55

Migration of myeloid cells	2.26	-0.45
Digestive system cancer	2.24	-0.88
Cell movement of macrophages	2.24	0.64
Growth of tumor	2.23	-0.40
Maturation of blood cells	2.21	-1.40
Influx of blood cells	2.21	-0.46
Homing of leukocytes	2.20	1.88
Binding of DNA	2.19	1.62
Interphase of gonadal cells	2.18	-1.21
Cell viability of tumor cells	2.18	1.15
Cell movement of phagocytes	2.17	1.11
Migration of cells	2.17	0.45
Activation of phagocytes	2.15	0.00
Abdominal cancer	2.15	-0.61
Alignment of chromosomes	2.14	0.00
Phosphorylation of protein	2.12	-0.19
Recruitment of leukocytes	2.12	-0.07
Adhesion of blood cells	2.10	0.44
Eosinophilia	2.10	-0.65
Glioma	2.07	-0.27
Cell movement	2.07	0.28
Binding of blood cells	2.06	0.36
Cell movement of antigen presenting cells	2.06	1.21
Binding of leukocytes	2.05	0.89
Chemotaxis of neutrophils	2.04	0.87
Gastrointestinal tract cancer	2.03	-1.19
Quantity of lymphoid cells	2.02	1.13
Maturation of cells	2.01	-0.97
Migration of granulocytes	2.01	-0.21
Transmigration of cells	2.00	0.34
Entry into interphase of oocytes	2.00	-1.00
Malignant neoplasm of large intestine	2.00	-1.00
Apoptosis of cancer cells	-2.28	1.33
Myelopoiesis of hematopoietic progenitor cells	-2.38	-1.67
Megakaryocytopoiesis	-2.55	-1.18
Hematopoiesis of bone marrow cells	-2.55	-1.18
Myelopoiesis of bone marrow	-2.55	-1.18

Hematopoiesis of bone marrow	-2.55	-1.18
